# Evolution of Ty1 copy number control in yeast by horizontal transfer and recombination

**DOI:** 10.1371/journal.pgen.1008632

**Published:** 2020-02-21

**Authors:** Wioletta Czaja, Douda Bensasson, Hyo Won Ahn, David J. Garfinkel, Casey M. Bergman

**Affiliations:** 1 Department of Biochemistry and Molecular Biology, University of Georgia, Athens, Georgia, United States of America; 2 Institute of Bioinformatics and Department of Plant Biology, University of Georgia, Athens, Georgia, United States of America; 3 Institute of Bioinformatics and Department of Genetics, University of Georgia, Athens, Georgia, United States of America; Cornell University, UNITED STATES

## Abstract

Transposable elements constitute a large fraction of most eukaryotic genomes. Insertion of mobile DNA sequences typically has deleterious effects on host fitness, and thus diverse mechanisms have evolved to control mobile element proliferation. Mobility of the Ty1 retrotransposon in *Saccharomyces* yeasts is regulated by copy number control (CNC) mediated by a self-encoded restriction factor derived from the Ty1 *gag* capsid gene that inhibits virus-like particle function. Here, we survey a panel of wild and human-associated strains of *S*. *cerevisiae* and *S*. *paradoxus* to investigate how genomic Ty1 content influences variation in Ty1 mobility. We observe high levels of mobility for a tester element with a *gag* sequence from the canonical Ty1 subfamily in permissive strains that either lack full-length Ty1 elements or only contain full-length copies of the Ty1’ subfamily that have a divergent *gag* sequence. In contrast, low levels of canonical Ty1 mobility are observed in restrictive strains carrying full-length Ty1 elements containing a canonical *gag* sequence. Phylogenomic analysis of full-length Ty1 elements revealed that Ty1’ is the ancestral subfamily present in wild strains of *S*. *cerevisiae*, and that canonical Ty1 in *S*. *cerevisiae* is a derived subfamily that acquired *gag* from *S*. *paradoxus* by horizontal transfer and recombination. Our results provide evidence that variation in the ability of *S*. *cerevisiae* and *S*. *paradoxus* strains to repress canonical Ty1 transposition *via* CNC is regulated by the genomic content of different Ty1 subfamilies, and that self-encoded forms of transposon control can spread across species boundaries by horizontal transfer.

## Author summary

Mobile DNA elements dominate the genomes of many eukaryotes, although their evolutionary dynamics are often not well understood. Here, we describe how the retrovirus-like Ty1 retrotransposon diversified in *Saccharomyces* yeasts in the face of a potent restriction factor encoded by the Ty1 *gag* gene. We show that mobility of a Ty1 tester element from *S*. *cerevisiae* with a *gag* sequence from the “canonical” Ty1 subfamily varies substantially across diverse yeast strains. Strains with full-length Ty1 elements carrying a canonical *gag* sequence in their genomes restrict tester Ty1 mobility while strains containing only elements from the Ty1’ subfamily do not, suggesting functional divergence among Ty1 subfamilies. Permissive strains lacking Ty1 elements with canonical *gag* can be converted to restrictive strains by introducing Ty1 elements with canonical *gag* into permissive genomes. Phylogenetic analyses show that Ty1’ is the ancestral subfamily in wild *S*. *cerevisiae* lineages, whereas canonical Ty1 is a derived subfamily found only in human-associated strains. Furthermore, the canonical Ty1 *gag* arose by recombination between an ancestral Ty1’ element and a Ty1 element horizontally transferred from *S*. *paradoxus*. Our results highlight how exchange of retrotransposon sequences across species can impact the repertoire and mobility of mobile DNA elements in eukaryotic genomes.

## Introduction

Retrotransposons are mobile genetic elements that transpose *via* an RNA intermediate and impact genome size, structure, function and molecular evolution in diverse eukaryotic lineages [1,2]. The budding yeast *Saccharomyces cerevisiae* is a powerful model organism for studying retrovirus-like long terminal repeat (LTR) retrotransposons, with many fundamental aspects of retrotransposon biology initially characterized in this species [3–5]. The complete sequencing of the yeast genome provided the first insight into organization and evolution of retrotransposons at the genomic scale [6–10]. More recently, advances in sequencing technologies and bioinformatics have provided unprecedented opportunities to investigate the evolutionary dynamics and consequences of transposition across yeast populations and species [11–20].

The current assembly of the *S*. *cerevisiae* S288c reference strain contains sequences from six families of LTR retrotransposons, four that are active (Ty1, Ty2, Ty3, and Ty4) and two that are inactive (Ty5 and Ty3_1p) [7,13]. At least 50 complete and over 400 partial Ty elements comprise 3.3% of the S288c reference assembly [7,13]. The abundance of complete or partial Ty elements and their solo LTR derivatives varies significantly between *S*. *cerevisiae* strains, where relatively high Ty content is observed in lab strains such as S288c relative to wild strains [12–14,21,22]. In general, full-length Ty element insertions are strain-specific or shared by only a few strains, while most solo LTR insertions and a few truncated “relic” elements are found at high allele frequency [13,14]. Additionally, some Ty elements in *S*. *cerevisiae* have distinct subfamilies (e.g. the Ty1’ and Ty1/2 subfamilies of Ty1) [7,8,23] or show evidence of recent horizontal transmission from other species (e.g. Ty2 and Ty3_1p) [13,22].

Ty1 is the most abundant retrotransposon family in the *S*. *cerevisiae* reference strain S288c (>30 full-length copies), and is both actively transcribed and transpositionally competent (reviewed in [4]). The structure and replication of Ty1 elements resembles that of retroviruses. Ty1 consists of two partially overlapping open reading frames–*gag* (*TYA*) and *pol* (*TYB*)–flanked by LTRs. mRNA from full-length Ty1 elements serves as a template for both reverse transcription and translation of the proteins necessary for retrotransposition: the Gag capsid protein, protease, integrase, and reverse transcriptase. Ty1 RNA is specifically packaged into virus-like particles (VLPs) and serves as the template for reverse transcription into linear cDNA, which subsequently is imported into the nucleus as a protein/DNA complex using a nuclear localization signal present on integrase. Ty1 preferentially integrates near genes transcribed by RNA Polymerase III through an association between integrase and Pol III-complexes [24,25].

Because of the deleterious effects of most transposition events, eukaryotic hosts have evolved effective mechanisms to restrict the mobility or expression of transposons including RNAi, DNA methylation, and APOBEC proteins [26,27]. Importantly, none of those systems operate natively in *S*. *cerevisiae* or its sister species *S*. *paradoxus* [28], two species that diverged 4–5 million years ago [29]. Instead, Ty1 mobility in *S*. *cerevisiae* and *S*. *paradoxus* is limited by a novel retroelement-directed restriction mechanism termed Copy Number Control (CNC) [21,30–33]. CNC is defined as a decrease in Ty1 mobility when additional copies of the Ty1 element are present in the genome. CNC is mediated by the Ty1 restriction protein p22, which is a truncated version of Gag encoded by internally-initiated Ty1 transcripts [32]. p22 interferes with a central function of the Ty1 capsid during VLP assembly and maturation, and thus is a potent self-encoded *trans*-dominant negative inhibitor of Ty1 retrotransposition [32,34,35]. Ty1 inhibition by p22 bears striking similarities to host-encoded restriction factors that inhibit retrovirus assembly or capsid uncoating [36].

To date, Ty1 CNC has been studied in a very limited number of genetic backgrounds: first in *S*. *paradoxus* strain 337 [30,32,37,38] and more recently in *S*. *cerevisiae* strain DJ12 [33]. Thus, it remains an open question at what level CNC operates in diverse lineages of *S*. *cerevisiae* and *S*. *paradoxus* that vary in their endogenous genomic Ty1 content. Here we use well-developed methods to measure mobility of a Ty1 tester element (called Ty1-H3) with a *gag* sequence from the “canonical” Ty1 subfamily in *S*. *cerevisiae* [39–41] across a diverse panel of *S*. *cerevisiae* and *S*. *paradoxus* strains. Our results reveal that Ty1-H3 mobility varies substantially among strains in both species. We show that “permissive” strains with high Ty1-H3 mobility can be converted to “restrictive” strains with low Ty1-H3 mobility by experimentally introducing multiple Ty1-H3 elements into permissive genomes, implying that permissive strains are competent to express Ty1 CNC. Additionally, we investigated the genomic basis of variation in Ty1-H3 mobility using whole genome PacBio long-read assemblies that yield complete sequence information of transposable elements in their native chromosomal locations [18,42,43]. By comparing Ty1 copy number and sequence composition with mobility frequency, we infer that restrictive strains in both *S*. *cerevisiae* and *S*. *paradoxus* contain full-length Ty1 elements with a canonical form of *gag*. In contrast, permissive strains either lack full-length Ty1 elements or only contain full-length elements from the Ty1’ subfamily that have a divergent *gag* sequence. Surprisingly, the reconstructed evolutionary history of full-length Ty1 elements in *S*. *cerevisiae* and *S*. *paradoxus* shows that the Ty1’ subfamily is the ancestral subfamily in *S*. *cerevisiae* found in wild lineages, while the canonical Ty1 family used in most functional studies is a highly-derived element found in human-associated strains. Furthermore, we discovered that the *gag* region of the canonical *S*. *cerevisiae* Ty1 element was acquired by horizontal transfer from an Old-World lineage of *S*. *paradoxus* followed by recombination onto a pre-existing ancestral Ty1’-like element. Our results demonstrate that intraspecific variation in the ability to repress transposition of elements from the canonical Ty1 subfamily in *S*. *cerevisiae* is a consequence of horizontal transfer of a CNC-competent *gag* gene from a closely-related yeast species.

## Results

### Ty1 restriction varies across diverse isolates of *S*. *cerevisiae* and *S*. *paradoxus*

Because Ty1 CNC is mediated by a self-encoded factor (p22) and dependent on Ty1 genomic copy number, we hypothesized that variation in endogenous Ty1 genomic content may influence the strength of Ty1 CNC across *Saccharomyces* strains. To address this possibility, we used Southern analysis to screen a set of genetically-tractable haploid derivatives of 25 *S*. *cerevisiae* strains and 27 *S*. *paradoxus* strains from the *Saccharomyces* Genome Resequencing Project (SGRP) for variation in their Ty1 content [12,44]. The probe for hybridization experiments in both species is derived from the *gag* region of the Ty1-H3 element (S1A Fig). Ty1-H3 is a full-length competent Ty1 element used in many pioneering studies on Ty1 structure and function that was isolated in *S*. *cerevisiae* as a His^+^ reversion mutant [45–47]. Because Ty1-H3 has played an important role in defining our understanding of Ty1 structure and function, we designate it as the “canonical” form of Ty1. However, as detailed below, Ty1-H3 shows evidence of recombination with the Ty1’ subfamily in its *pol* region and thus the Ty1-H3 is not a “pure” representative of the canonical Ty1 subfamily. Southern analysis revealed substantial diversity across both *S*. *cerevisiae* and *S*. *paradoxus* strains in the number of Ty1 elements that share strong sequence similarity to Ty1-H3 *gag* (S1B Fig and S1C Fig), with most strains having fewer Ty1 elements than the *S*. *cerevisiae* reference strain S288c. These results also revealed several strain genomes where we failed to detect hybridization with the Ty1-H3 *gag* probe, consistent with the existence of multiple “Ty1-less” strains that lack full-length elements in *S*. *cerevisiae* and *S*. *paradoxus* [16,18,21,37].

Next, we selected a diverse panel of seven *S*. *cerevisiae* and three *S*. *paradoxus* SGRP strains with distinct Ty1 hybridization patterns (S1B Fig and S1C Fig) to test for variation in the frequency of mobility using a Ty1-H3 tester element marked with a *his3-AI* indicator gene [39]. We performed Ty1 mobility assays in the seven *S*. *cerevisiae* strains (S288c, Y12, DBVPG6044, UWOPS83-787.3, YPS606, UWOPS05-227.2, L-1374) by introducing a *URA3*-based centromere plasmid containing a competent Ty1*his3-AI* element (pOy1) into haploid *MATα ho*::*HygroMX ura3*::*KanMX his3-Δ200hisG* SGRP strains. The frequency of His^+^ colony formation in this assay detects Ty1 mobility events from either *de novo* retrotransposition events or insertion events from a minor pathway where Ty1 cDNA undergoes homologous recombination with genomic or plasmid-borne Ty1 sequences [48]. For the three *S*. *paradoxus* strains tested (CBS432, N-44, YPS138), deletion of *HIS3* could not be achieved efficiently and thus Ty1 mobility assays were performed by first replacing the *KanMX* gene inserted at the *URA3* locus with *NatMX* in haploid *MATα* SGRP strains, then introducing a reporter plasmid containing Ty1*neo-AI* (pBDG954) into the resulting *MATα ho*::*HygroMX ura3*::*NatMX* strains. The appearance of G418-resistant colonies in these *S*. *paradoxus* strains is a readout for retromobility that can be monitored by qualitative or quantitative assays similar to Ty1*his3-AI* reporter system [39–41]. These experiments revealed >50-fold differences in the mobility of Ty1-H3 across strains within both *S*. *cerevisiae* and *S*. *paradoxus* (Table 1). In both species, we observed “restrictive” strains with very low levels of Ty1-H3 mobility (*S*. *cerevisiae*: S288c, Y12, and DBVPG6044; *S*. *paradoxus*: CBS432, N-44). Likewise, we observed “permissive” strains in both species with Ty1-H3 mobility frequencies which were more than an order of magnitude higher than restrictive strains (*S*. *cerevisiae*: UWOPS05-787.3, YPS606, UWOPS05-227.2, and L1374; *S*. *paradoxus*: YPS138).

**Table 1:** Canonical Ty1-H3 mobility in a diverse panel of *S*. *cerevisiae* and *S*. *paradoxus* strains.

Strain	Species	Source	Origin	Ty1*his3-AI* x10^-6^ [S.D.]	Ty1*neo-AI* x10^-7^ [S.D.]	Ty1-H3 mobility phenotype
S288c	*S*. *cerevisiae*	Lab	N. America	1.6 [0.2]	N.D.	restrictive
DBVPG6044	*S*. *cerevisiae*	Bili Wine	West Africa	1.0 [0.17]	N.D.	restrictive
Y12	*S*. *cerevisiae*	Sake	Japan	5.6 [0.5]	N.D.	restrictive
UWOPS83-787.3	*S*. *cerevisiae*	Wild	Bahamas	78 [5.5]	N.D.	permissive
YPS606	*S*. *cerevisiae*	Wild	N. America	157 [13]	N.D.	permissive
UWOPS05-227.2	*S*. *cerevisiae*	Wild	Malaysia	458 [86]	N.D.	permissive
L-1374	*S*. *cerevisiae*	Wine	Chile	635 [79]	N.D.	permissive
CBS432	*S*. *paradoxus*	Wild	Europe	N.D.	<0.6	restrictive
N-44	*S*. *paradoxus*	Wild	Far East Asia	N.D.	0.9 [0.8]	restrictive
YPS138	*S*. *paradoxus*	Wild	N. America	N.D.	31 [7]	permissive

Ty1 mobility frequency is defined as the number revertant colonies (His^+^ Ura^+^ colonies for Ty1*his3*-AI or G418^R^ colonies for Ty1*neo*-AI) colonies divided by the number of Ura^+^ colonies per ml of culture. Standard deviations were calculated from the number Ty1 mobility events detected per 1 ml culture. Because of differences in reporter constructs and selection systems, mobility frequencies can be compared across strains within species, but not across species. S.D. = standard deviation. N.D. = not determined.

Variation in Ty1 mobility across *Saccharomyces* strains could result from variation in the strength of CNC conferred by endogenous Ty1 elements or other differences in host genetic background that render some strains incapable of expressing Ty1 CNC. To determine whether Ty1 mobility in permissive strains is due to host backgrounds that are unable to manifest Ty1 CNC, we over-expressed Ty1-H3 from a plasmid to “populate” the genomes of three permissive *S*. *cerevisiae* strains (UWOPS05-227.2, L1374, and YPS606) with >8 Ty1-H3 elements, as estimated by Southern analysis (see Materials and Methods for details). As shown previously for *S*. *paradoxus* strain 337 [30], we observed a >60-fold decrease in Ty1-H3 mobility in three *S*. *cerevisiae* strains populated with multiple Ty1-H3 elements when compared with their respective native parental strains (Table 2), with Ty1-H3 mobility in populated strains being on the same order as other native strains with restrictive phenotypes. We note that mobility data for native strains in Table 2 were from an independent set of experiments done in parallel with populated strains and thus differ slightly from the data in Table 1 for the same native strains. The ability for all three permissive strains tested to become restrictive with the addition of full-length copies of Ty1-H3 indicates that the genetic background of permissive strains is competent to express canonical Ty1 CNC and is consistent with the hypothesis that genomic Ty1 content plays an important role in shaping variation in Ty1 mobility among yeast strains.

**Table 2:** Canonical Ty1-H3 mobility in natively permissive *S*. *cerevisiae* strains populated with canonical Ty1-H3 elements.

Strain	Species	Ty1*his3-AI* x10^-6^ [SD]	Ty1-H3 mobility phenotype
YPS606	*S*. *cerevisiae*	164 [8.5]	permissive
YPS606 + 8 canonical Ty1 elements	*S*. *cerevisiae*	2.4 [0.12]	restrictive
UWOPS05-227.2	*S*. *cerevisiae*	529 [124]	permissive
UWOPS05-227.2 + 17 canonical Ty1 elements	*S*. *cerevisiae*	1 [0.25]	restrictive
L-1374	*S*. *cerevisiae*	461 [163]	permissive
L-1374 + 20 canonical Ty1 elements	*S*. *cerevisiae*	3 [0.35]	restrictive

Canonical Ty1 copy number in populated strains was estimated by Southern blot analysis as described previously [30].

### The presence of full-length Ty1 elements is not sufficient to restrict Ty1-H3 mobility

To determine if variation in Ty1 mobility is influenced by the copy number or sequence of endogenous Ty1 elements, we generated ~100x whole-genome shotgun PacBio datasets and assembled genome sequences for the seven *S*. *cerevisiae* strains assayed for Ty1 mobility. We integrated data from our *S*. *cerevisiae* PacBio assemblies with similar high quality PacBio genome assemblies from Yue *et al*. [18] for the three strains of *S*. *paradoxus* with mobility data in our study (CBS432, N-44, YPS138). PacBio assemblies typically reconstructed complete chromosomes in single contigs (with the exception of chromosome XII which was broken at the highly repeated rDNA locus) and thus provide an essentially-complete catalogue of Ty content in yeast genomes. We identified Ty elements in these ten PacBio assemblies using a RepeatMasker-based strategy that classifies Ty elements as full-length, truncated, or solo LTR sequences based on the completeness of internal sequences in each predicted element (see Materials and Methods for details). Although our focus is on Ty1, we annotated all Ty families in these genomes to avoid potential misidentification, and because the similarity of solo LTRs from Ty1 and Ty2 does not allow their unambiguous assignment to either family (see also Yue *et al*. [18]). Predicted numbers of full-length, truncated, or solo LTR sequences for Ty1 can be found in Table 3 and for all Ty families in S1 File. We focused on full-length elements in our analysis since they are most likely to have the complete set of functional sequences required for Ty1 gene expression and transposition.

**Table 3:** Ty1 content in a diverse panel of *S*. *cerevisiae* and *S*. *paradoxus* strains with Ty1 mobility phenotypes.

Strain	Species	Ty1 mobility phenotype	Full-length Ty1	Truncated Ty1	Ty1/Ty2 solo LTRs	Full length with canonical Ty1 *gag*	Full length with Ty1’ *gag*	Truncated with canonical Ty1 *gag*	Truncated with canonical Ty1’ *gag*
S288c	*S*. *cerevisiae*	restrictive	38	2	161	35	3**	1	1
DBVPG6044	*S*. *cerevisiae*	restrictive	19	5	269	19	0	1	1
Y12	*S*. *cerevisiae*	restrictive	19	2	158	8*	11	0	1
UWOPS83-787.3	*S*. *cerevisiae*	permissive	7	3	158	0	7	0	1
YPS606	*S*. *cerevisiae*	permissive	3	2	147	0	3	0	1
UWOPS05-227.2	*S*. *cerevisiae*	permissive	0	2	185	0	0	0	1
L-1374	*S*. *cerevisiae*	permissive	0	1	155	0	0	0	1
CBS432	*S*. *paradoxus*	restrictive	8	4	264	8	0	1	0
N-44	*S*. *paradoxus*	restrictive	2	3	235	2	0	0	0
YPS138	*S*. *paradoxus*	permissive	0	1	232	0	0	0	0

Asterisks represent inclusion of two elements (*: Y12_f109; **: S288c_f486) that are recombinant between Ty1’ and canonical Ty1 *gag* and are classified according to the group from which the majority of their *gag* sequence is derived. Classification of Ty1 *gag* type for truncated elements is only for elements that retain sequences with homology to Ty1-H3 *gag* in the p22 region.

The total number of full-length Ty1 elements varies substantially across the ten yeast strains with mobility data in our sample (Table 3). *S*. *cerevisiae* strains can have high (S288c), intermediate (DBVPG6044 and Y12), or low (UWOPS05-787.3, and YPS606) Ty1 copy number, or are Ty1-less (UWOPS05-227.2 and L1374). *S*. *paradoxus* strains either have low copy number (CBS432 and N-44) or are Ty1-less (YPS138). All strains contain truncated Ty1 elements and Ty1-like solo LTRs, indicating that Ty1 was present in the ancestor of all strains in both species and that Ty1-less strains arose by multiple independent losses of full-length Ty1 elements, presumably by LTR-LTR recombination. Integrating genomic Ty1 content with mobility data, we observe that all restrictive strains contain multiple full-length copies of Ty1 elements (*S*. *cerevisiae*: S288c, Y12, DBVPG6044; *S*. *paradoxus*: CBS432, N-44), consistent with the expectation that repression of Ty1 mobility is mediated by Ty1 CNC. Also consistent with predictions of the Ty1 CNC mechanism, Ty1-less strains are permissive (*S*. *cerevisiae*: UWOPS05-227.2, L1374; *S*. *paradoxus*: YPS138). However, we observed two permissive strains in *S*. *cerevisiae* that unexpectedly contained full-length Ty1 elements (UWOPS83-787.3, YPS606). These results indicate that variation in the frequency of Ty1 mobility across strains cannot be explained by a simple model whereby the presence of a full-length Ty1 element in the genome is sufficient to confer a restrictive phenotype.

### Recombination occurs among canonical Ty1 and Ty1’ subfamilies in *S*. *cerevisiae*

**Fig 1:**
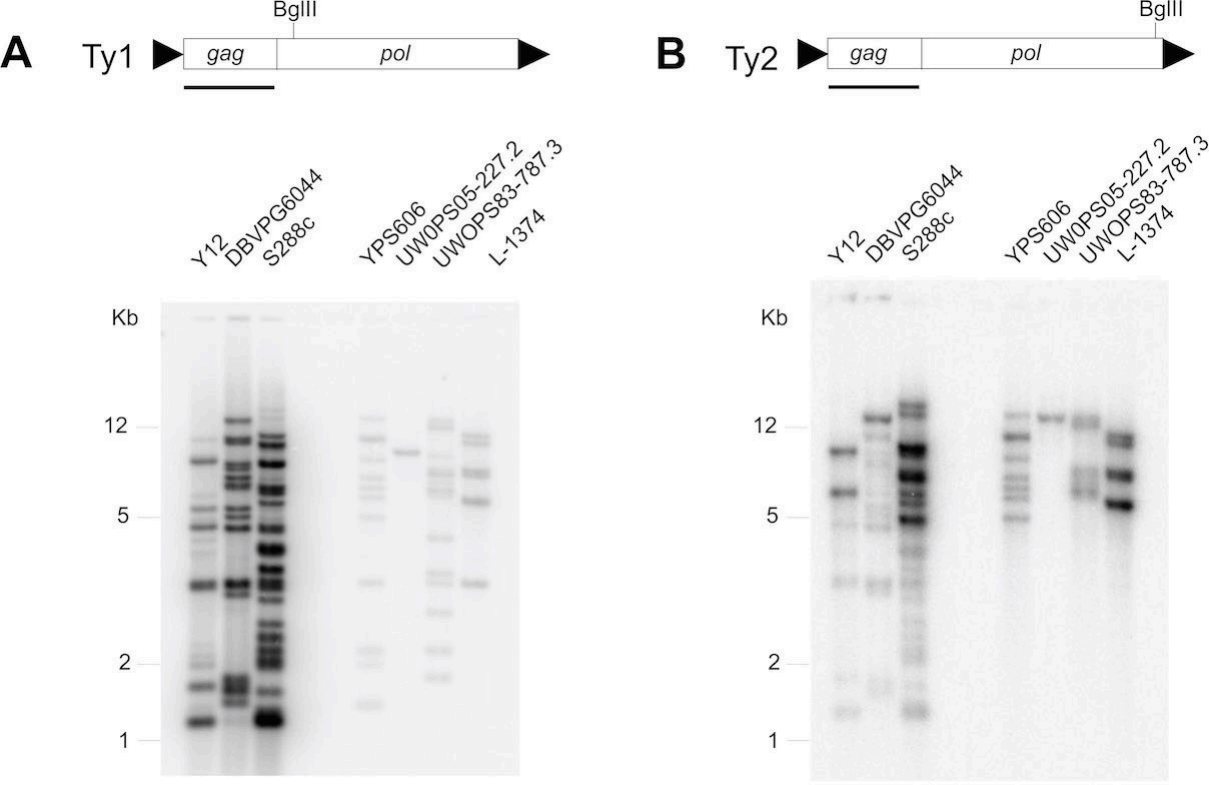
Southern blots of canonical Ty1-H3 and Ty2 *gag* hybridized to *S*. *cerevisiae* strains with Ty1-H3 mobility phenotypes. Southern blot results using radiolabeled (**A**) Ty1 and (**B**) Ty2 *gag* probes for *S*. *cerevisiae* strains with mobility data. Restrictive strains are shown on the left, and permissive strains are shown on the right of each Southern blot. Note that permissive strains typically contain weakly hybridizing bands to Ty1 below 5 kb in length that are not caused by cross-hybridization to Ty2. Schematics of the Ty1-H3 and Ty2-917 elements, respectively, showing the *gag* and *pol* open reading frames (rectangles) and LTRs (arrowheads) are shown above each Southern blot. The probes used for Southern blots were obtained from the *gag* gene of each element (underlined) and are 1,162 bp and 1,158 bp in length for Ty1-H3 and Ty2-917, respectively. The complete Ty1-H3 element is 5918 bp in length and the complete Ty2-917 element is 5960 bp in length. The locations of the BglII restriction sites within Ty1-H3 and Ty2-917 *pol* regions are shown above the schematics. Identical membranes with the same DNA samples were hybridized in panels (A) and (B).

The two exceptional *S*. *cerevisiae* permissive strains that had full-length Ty1 elements detected in their PacBio assemblies (UWOPS05-787.3 and YPS606) displayed multiple bands with weak hybridization to the Ty1 *gag* probe by Southern blot analysis (Fig 1A). Some, but not all, of these weak Ty1 bands could be explained by cross-hybridization with Ty2 (Fig 1B). This observation suggested the possibility of divergent Ty1 sequences in these genomes such as the Ty1’ subfamily that is known to differ from the canonical Ty1 subfamily in its *gag* region [7]. To determine if the presence of a variant Ty1 subfamily could potentially explain the observation of permissive strains with full-length Ty1 elements, we extracted and aligned all full-length Ty1 elements from the PacBio assemblies of the ten *S*. *cerevisiae* and *S*. *paradoxus* strains for which we had mobility data, then clustered full-length Ty1 elements based on sequence similarity. We included the Ty1-H3 tester element used in our mobility assays and used a distance-based clustering approach (Neighbor Joining) in this analysis, since our goal was to identify potential Ty1 subfamilies that could explain variation in Ty1-H3 mobility across strains, not to infer the detailed evolutionary history of Ty1 in these species.

**Fig 2:**
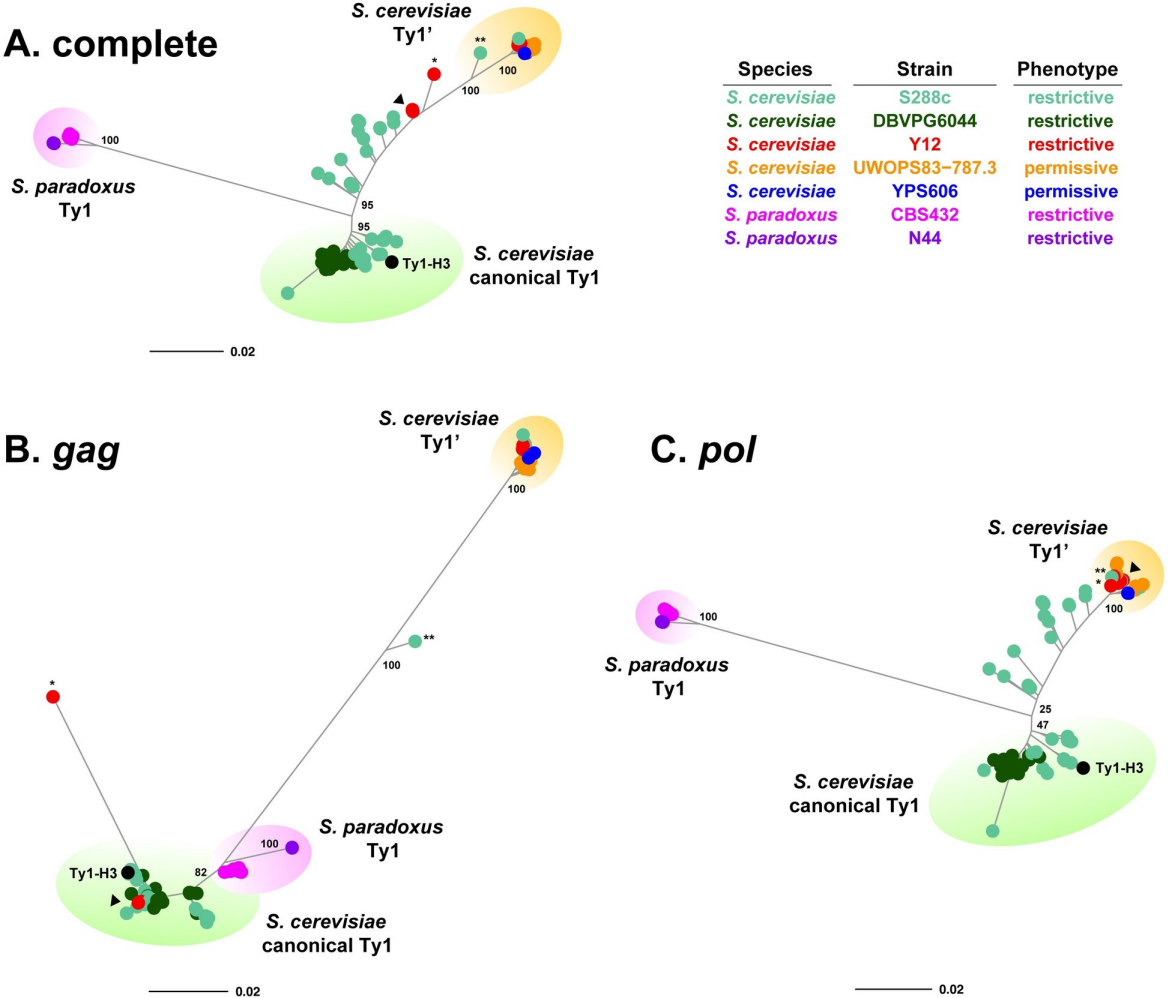
Clustering of sequences from full-length Ty1 elements in *Saccharomyces* strains with Ty1-H3 mobility phenotypes. Unrooted consensus trees for full-length elements based on (**A**) complete Ty1 sequences, (**B**) the Ty1 *gag* gene region only, and (**C**) the Ty1 *pol* gene region only. Trees were constructed using the BioNJ clustering algorithm. Numbers at key nodes represent bootstrap support based on 100 replicates. The scale bar for branch lengths is in units of substitutions per site. The Ty1-H3 tester element used in mobility assays is shown in black. Recombinant elements between Ty1’ and canonical Ty1 within *gag* are starred (*Y12_f109; **: S288c_f486). Seven closely related “mosaic” Y12 elements that are recombinants between canonical Ty1 and Ty1’ are labeled with an arrowhead. We note that in some cases filled circles can represent multiple identical or nearly identical sequences (e.g. seven closely related Y12 elements, arrowhead).

Clustering of complete Ty1 sequences revealed a well-supported long branch separating *S*. *cerevisiae* elements from those in *S*. *paradoxus* (Fig 2A). In *S*. *cerevisiae*, two major clusters of Ty1 elements are observed. One cluster corresponds to the canonical Ty1 subfamily as defined by the presence of the Ty1-H3 tester element in this cluster (green background, Fig 2A). Two strains have full-length elements in the canonical Ty1 cluster (S288c and DBVPG6044). The other major *S*. *cerevisiae* cluster (found in S288c, Y12, UWOPS83-787.3, and YPS606) contains three elements previously defined as the Ty1’ subfamily in S288c by Kim *et al*. [7] (orange background, Fig 2A). The canonical Ty1 and Ty1’ clusters are separated by a long internal branch containing multiple short branches leading to individual Ty1 elements or small groups of closely-related Ty1 elements from Y12 and S288c. Inspection of our multiple sequence alignment revealed that one of these elements (Y12_f109; single asterisk, Fig 2A) is in fact a recombinant element derived from an exchange event between canonical Ty1 and Ty1’ sequences within the *gag* region (S2A Fig). A second recombinant in *gag* between canonical Ty1 and Ty1’ sequences was also found in our dataset (S288c_f486; double asterisk, Fig 2A; S2B Fig), which previously was classified as a divergent Ty1’ element by Kim *et al*. [7] (SGD: YNLCTy1-1). Sliding window analysis showed that these two recombinant elements are essentially Ty1’ elements with fragments of canonical Ty1 sequence in their *gag* regions (S2A Fig and S2B Fig). Full-length elements from the canonical Ty1 or Ty1’ subfamilies are not found in *S*. *paradoxus*, implying that both of these subfamilies are specific to *S*. *cerevisiae*.

Because previous work shows that Ty1 *gag* and *pol* genes have different evolutionary histories in *S*. *cerevisiae* [14,23], we next clustered full-length Ty1 elements on the basis of their *gag* (Fig 2B) and *pol* (Fig 2C) sequences separately. Clustering of elements based on Ty1 *gag* revealed a discordant topology relative to that from complete sequences, with no long branch separating *S*. *paradoxus* and *S*. *cerevisiae* and essentially no elements on the long internal branch separating the canonical Ty1 and Ty1’ clusters (except the recombinant S288c_f486 element noted above). In the *gag* tree, *S*. *paradoxus* elements unexpectedly clustered closely with *S*. *cerevisiae* canonical Ty1 elements, indicating a previously-unreported similarity between *S*. *paradoxus* Ty1 *gag* and *S*. *cerevisiae* canonical Ty1 *gag* (see below). Y12 and S288c elements found on the long branch between canonical Ty1 and Ty1’ clusters in the complete sequence tree (Fig 2A) cluster in the canonical group in the *gag* tree (Fig 2B), indicating that these elements all have a canonical Ty1 type *gag* gene. The recombinant Y12_f109 element noted above clusters with the canonical Ty1 group (single asterisk, Fig 2B) since the majority of its *gag* gene is canonical Ty1 but is found on a very long unique branch due to the presence of Ty1’ sequences in the 5’ part of its *gag* (S2A Fig). Aside from these two elements with evidence of recombination in *gag*, all full-length Ty1 elements are found in two main groups separated by substantial sequence divergence in their *gag* region: (i) elements with a canonical Ty1 type *gag* found in *S*. *cerevisiae* and *S*. *paradoxus*, and (ii) elements with a Ty1’ type *gag* found only in *S*. *cerevisiae*.

Clustering of Ty1 *pol* (Fig 2C) revealed a topology similar to that of complete Ty1 sequences (Fig 2A) with two notable exceptions. First, the two elements that are recombinant in *gag* (Y12_f109 and S288c_f486) are both found within the Ty1’ cluster in their *pol* regions, consistent with these elements being predominantly Ty1’ except for parts of their *gag* genes (S2A Fig and S2B Fig). Second, the seven closely-related Y12 elements found on the long internal branch in the complete tree (arrowhead, Fig 2A) cluster in the Ty1’ group in the *pol* tree (arrowhead, Fig 2C). This observation, in addition to the fact that these seven Y12 elements have a canonical Ty1 *gag* (arrowhead, Fig 2B), implies that they are recombinants between the canonical Ty1 and Ty1’ subfamilies with an exchange event somewhere near the boundary of *gag* and *pol* (see below). Phylogenetic network analysis revealed that the remaining S288c elements on the long internal branch in the *pol* tree, plus additional elements in the canonical Ty1 cluster (including the Ty1-H3 tester element), are recombinants between the canonical Ty1 and Ty1’ subfamilies within the *pol* region (S3 Fig). Canonical Ty1 elements from DBVPG6044, however, show no evidence of recombination in *pol* (or *gag*) and therefore best represent “pure” canonical Ty1 elements. Thus, all *S*. *cerevisiae* elements found on the long internal branch between canonical Ty1 and Ty1’ subfamilies in the complete sequence tree exhibit recombination between subfamilies within *gag*, near the boundary of the *gag* and *pol*, or within *pol*.

### *Saccharomyces* strains that restrict Ty1-H3 mobility encode full-length elements with canonical Ty1 *gag*

We next attempted to interpret variation in Ty1-H3 mobility at the strain level with genomic Ty1 content partitioned by the type of *gag*–canonical Ty1 or Ty1’–encoded by full-length elements. The rationale for this analysis is based on p22 being encoded in the C-terminal half of *gag*, and recombination between the canonical Ty1 and Ty1’ subfamilies precluding straightforward classification at the complete element level. We classified *S*. *paradoxus* Ty1 elements as having canonical Ty1 type *gag* because of the close clustering with *S*. *cerevisiae* canonical Ty1 sequences (Fig 2B). This analysis revealed that restrictive strains from both *S*. *cerevisiae* and *S*. *paradoxus* contain one or more full-length elements that encode a canonical Ty1 type *gag* (*S*. *cerevisiae*: S288c, DBVPG6044 and Y12; *S*. *paradoxus*: CBS432 and N-44) (Table 3). Conversely, permissive strains only have full-length elements that encode Ty1’ *gag* (*S*. *cerevisiae*: UWOPS83-787.3 and YPS606) or lack full-length Ty1 elements altogether (*S*. *cerevisiae*: UWOPS05-227.2, L1374; *S*. *paradoxus*: YPS138). These results suggest that the ability to restrict mobility of the Ty1-H3 tester element requires a full-length Ty1 element in the genome with sufficient sequence similarity to canonical Ty1 *gag*. Because the two restrictive strains with recombinants between canonical Ty1 and Ty1’ in *gag* (Y12 and S288c) each also have full-length elements with a complete canonical Ty1 *gag* gene, the presence of these two recombinants does not alter this general conclusion. The dependency of Ty1-H3 mobility on the presence of full-length Ty1 elements with canonical *gag* appears to hold quantitatively as well. Ty1-H3 mobility and the number of full-length Ty1 elements that have a canonical Ty1 *gag* are negatively correlated across *S*. *cerevisiae* strains (rho = -0.8669214; p-value = 0.01154), consistent with the general hypothesis that increasing genomic canonical Ty1 *gag* content increases the strength of canonical Ty1 CNC [30].

**Fig 3:**
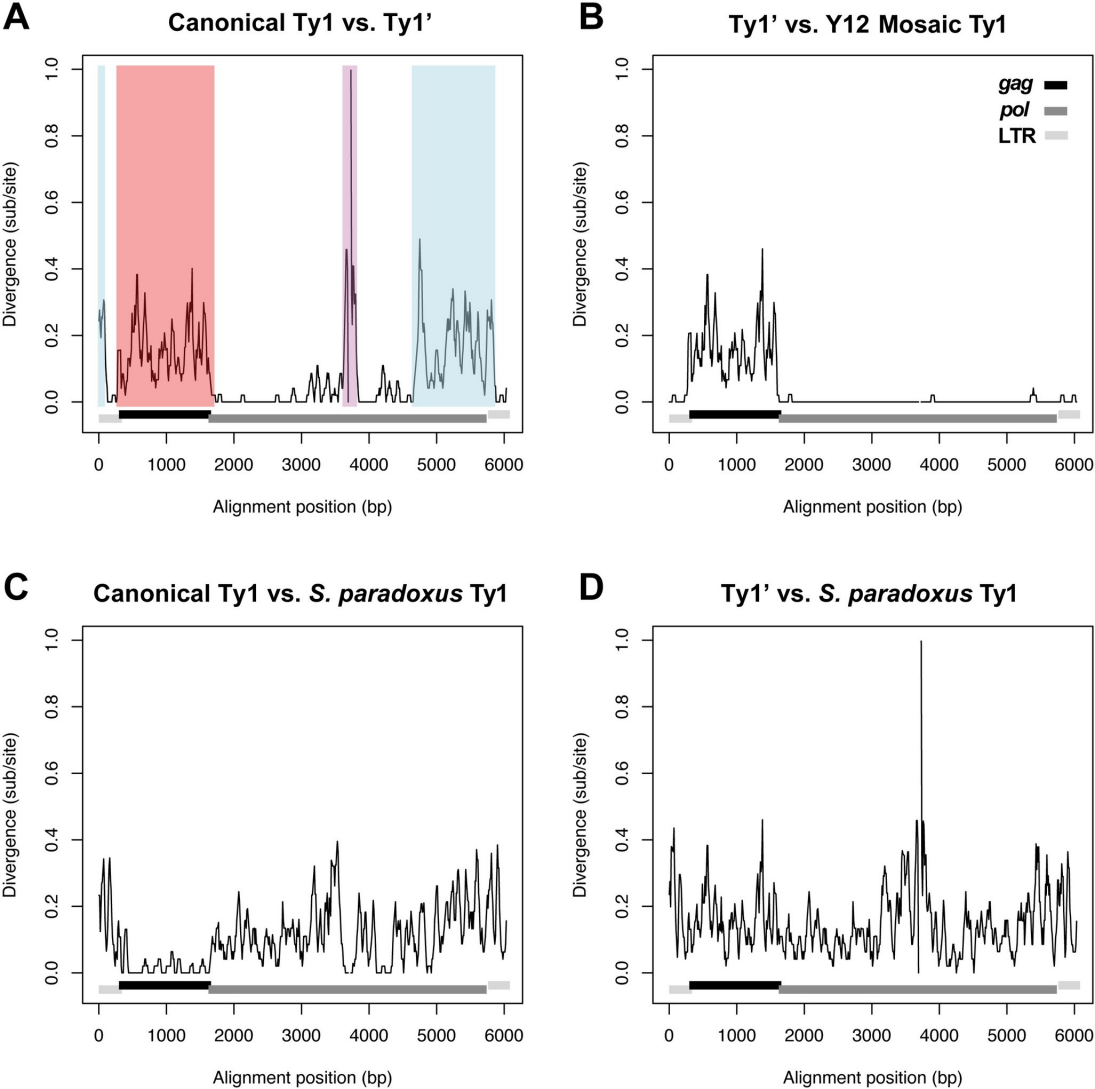
Sequence divergence between Ty1 elements in *S*. *cerevisiae* and *S*. *paradoxus*. Sliding window analysis of pairwise sequence divergence between (**A**) *S*. *cerevisiae* canonical Ty1 vs. *S*. *cerevisiae* Ty1’, (**B**) *S*. *cerevisiae* Ty1’ vs. *S*. *cerevisiae* mosaic Ty1 (found in the restrictive Y12 strain), (**C**) *S*. *cerevisiae* canonical Ty1 vs. *S*. *paradoxus* Ty1, and (**D**) *S*. *cerevisiae* Ty1’ vs. *S*. *paradoxus* Ty1. Functional regions of the Ty1 element are annotated as follows: LTRs (light grey); *gag* (black); *pol* (dark grey). Colored regions in (**A**) depict parts of Ty1 showing high divergence between *S*. *cerevisiae* canonical Ty1 vs. *S*. *cerevisiae* Ty1’. Blue: divergence in LTRs and *pol* caused by recombination between canonical Ty1 and Ty2 (see also [8,23] and S5 Fig); red: divergence in *gag* caused by recombination between canonical Ty1 and *S*. *paradoxus* Ty1; purple: divergence in *pol* caused by recombination between canonical Ty1 and *S*. *paradoxus* Ty1. Elements used are: DBVPG6044_f486 (*S*. *cerevisiae* pure canonical Ty1); Y12_f208 (*S*. *cerevisiae* pure Ty1’); Y12_f429 (*S*. *cerevisiae* mosaic Ty1); CBS432_f139 (European *S*. *paradoxus* Ty1). Divergence measured in substitutions per site was calculated using a Kimura 2-parameter model in overlapping 50 bp windows with a 10 bp step size.

One confounding factor to the interpretation that having a full-length element with a canonical Ty1 *gag* is required to confer the restrictive phenotype is that there is substantial sequence divergence between canonical Ty1 and Ty1’ not only in *gag* (red, Fig 3A) but also in parts of both LTRs and *pol* (blue and purple, Fig 3A). The impact of divergence in *gag* can be separated from other changes between canonical Ty1 and Ty1’ using data from the restrictive strain Y12. Full-length elements in Y12 are either “pure” Ty1’ (elements from the Ty1’ subfamily that show no evidence of recombination with the canonical Ty1 subfamily) or “mosaic” Ty1 (elements that are divergent from Ty1’ in *gag* like pure canonical elements but are otherwise nearly identical to Ty1’ in their LTRs and *pol* regions) (Fig 3B). Remarkably, mosaic Ty1 elements in Y12 have recombination breakpoints that coincide almost precisely with the *gag* open reading frame (ORF) (Fig 3B), which explains the discordant placement of these elements when clustered by *gag* versus *pol* (arrowheads, Fig 2B vs. Fig 2C). Thus, the main difference in Ty1 content between Y12 and other strains that carry only pure Ty1’ elements (i.e. UWOPS83-787.3 and YPS606) is the presence of mosaic Ty1 elements in Y12 that encode a canonical Ty1 *gag*. Given that other *S*. *cerevisiae* strains carrying only pure Ty1’ elements have a permissive phenotype (i.e. UWOPS83-787.3 and YPS606), the restrictive phenotype in Y12 suggests that having canonical Ty1 *gag* in mosaic Ty1 elements is sufficient (and that canonical LTRs and *pol* are not required) to restrict Ty1-H3 mobility.

How genomic Ty1 content influences mobility phenotypes is also complicated by the existence of truncated Ty1 elements which may contain functional p22 sequences. To address this, we aligned truncated Ty1 elements to the Ty1-H3 tester element to identify truncated elements that have the potential to code for p22. All *S*. *cerevisiae* strains with mobility data contain truncated Ty1 elements that span the p22 region of *gag* (Table 3, S4 Fig). Two *S*. *cerevisiae* strains (S288c, L-1374) share a truncated Ty1 element found on chromosome IV that includes all of *gag* plus the 5’ LTR and some of *pol*. Likewise, five *S*. *cerevisiae* strains (DBVPG6044, Y12, UWOPS83-787.3, YPS606, UWOPS05-227.2) share a common truncated Ty1 element found at a different location on chromosome IV that includes nearly all of *gag* but no other parts of Ty1. Additionally, two *S*. *cerevisiae* strains (S288c and DBVPG6044) have strain-specific truncated elements that also span the p22 region of *gag*. By integrating these truncated elements into the multiple sequence alignment of *gag* sequences from a diverse panel of yeast strains reported in Bleykastens-Grosshans *et al*. [14], we confirmed that the two different truncated Ty1 loci found on chromosome IV shared by multiple *S*. *cerevisiae* strains are the same as two high-frequency “relics” related to the Ty1’ subfamily reported by Bleykastens-Grosshans *et al*. [14]. The remaining two strain-specific *S*. *cerevisiae* truncated Ty1 elements that span the p22 region are derived from the canonical Ty1 lineage. All truncated Ty1 elements that span the p22 region in *S*. *cerevisiae* have the capacity to encode an ORF for p18, the proteolytically processed form of p22 that is also capable of restricting Ty1 transposition [32]. In *S*. *paradoxus*, only one strain (CBS432) has a truncated element with *gag* sequences that span the p22 region, but the truncated Ty1 element in this strain does not encode a complete ORF for p18/p22. From these data, we conclude that presence of truncated elements with canonical Ty1 *gag* may contribute to the restrictive mobility phenotypes of S288c and DBVPG6044, and that the presence of truncated elements with Ty1’ *gag* does not inhibit the restrictive phenotypes in S288c, DBVPG6044, and Y12. Conversely, four permissive *S*. *cerevisiae* strains that lack full-length elements with canonical Ty1 *gag* (UWOPS83-787.3, YPS606, UWOPS05-227.2 and L-1374) only contain truncated elements from the Ty1’ subfamily, and thus our conclusion that having full-length elements with a canonical Ty1 *gag* is required to confer the restrictive phenotype is unaltered by the presence of truncated elements with Ty1’ *gag* sequences in the genomes of these strains.

### Canonical *S*. *cerevisiae* Ty1 *gag* was recently acquired from *S*. *paradoxus* by horizontal transfer

Clustering of Ty1 sequences from strains with mobility data revealed a surprising similarity between the *gag* sequences of full-length elements from the *S*. *cerevisiae* canonical Ty1 cluster and *S*. *paradoxus* (Fig 2B). Sliding window divergence analysis revealed that the regions of high divergence between canonical *S*. *cerevisiae* Ty1 and *S*. *cerevisiae* Ty1’ in *gag* (red, Fig 3A) and the middle part of *pol* (purple, Fig 3A) correspond exactly to regions of high sequence similarity between canonical *S*. *cerevisiae* Ty1 and *S*. *paradoxus* Ty1 (Fig 3C). High sequence similarity is not observed in the corresponding regions between *S*. *cerevisiae* Ty1’ and *S*. *paradoxus* Ty1 (Fig 3D). These results suggest that the extreme divergence between canonical Ty1 and Ty1’ in *gag* may have resulted from horizontal transfer of a *S*. *paradoxus* Ty1 element and recombination with an ancestor of the *S*. *cerevisiae* canonical Ty1 subfamily after divergence of the canonical Ty1 and Ty1’ lineages and that the Ty1’ subfamily represents the ancestral state in *S*. *cerevisiae*.

To further investigate this putative horizontal transfer event and the ancestral state of the Ty1 family in *S*. *cerevisiae*, we reconstructed the phylogenetic history of *gag* and *pol* sequences from full-length Ty1 elements using an expanded set of *S*. *cerevisiae* and *S*. *paradoxus* strains with essentially-complete PacBio assemblies. This expanded dataset includes the ten strains analyzed above, plus six *S*. *cerevisiae* strains with diverse geographic origins (SK1, YPS128, UWOPS03-461.4, DBVPG6765, Sb-biocodex, Sb-unique28) and two New World *S*. *paradoxus* strains (UFRJ50816, UWOPS91-917.1) that have publicly-available PacBio data [18,42]. Additionally, we generated and included PacBio assemblies for three *S*. *cerevisiae* strains isolated from ancestral oak habitats in North America, Europe and Japan (SDO2s1, ZP568s1, ZP655.1A) to better sample Ty1 content in geographically-diverse wild *S*. *cerevisiae* lineages [49,50]. Ty1 sequences from PacBio assemblies of two *S*. *jurei* strains (NCYC 3947, NCYC 3962) reported in Naseeb *et al*. [43] were used to root trees and polarize changes on the *S*. *cerevisiae* and *S*. *paradoxus* lineages. Ty elements in these genomes were detected as described above and Ty content for all strains can be found in S1 File. Several strains in the expanded dataset in addition to those noted above were found to be Ty1-less and therefore are not represented in these trees: *S*. *cerevisiae* Wine/European (DBVPG6765, Sb-biocodex, Sb-unique28), *S*. *cerevisiae* Malaysian (UWOPS05-227.2, UWOPS03-461.4) and *S*. *paradoxus* N. America (YPS138). For this analysis, trees were generated using maximum likelihood so that ancestral states could be reconstructed for the *gag* gene. Two recombinants in *gag* noted above (S288c_f486 and Y12_f109) were excluded from this analysis since their inclusion distorted ancestral state reconstruction. Annotated trees for *gag* and *pol* with element identifiers can be found in S6 Fig, and tree files for *gag* and *pol* can be found in S2 and S3 Files, respectively.

**Fig 4:**
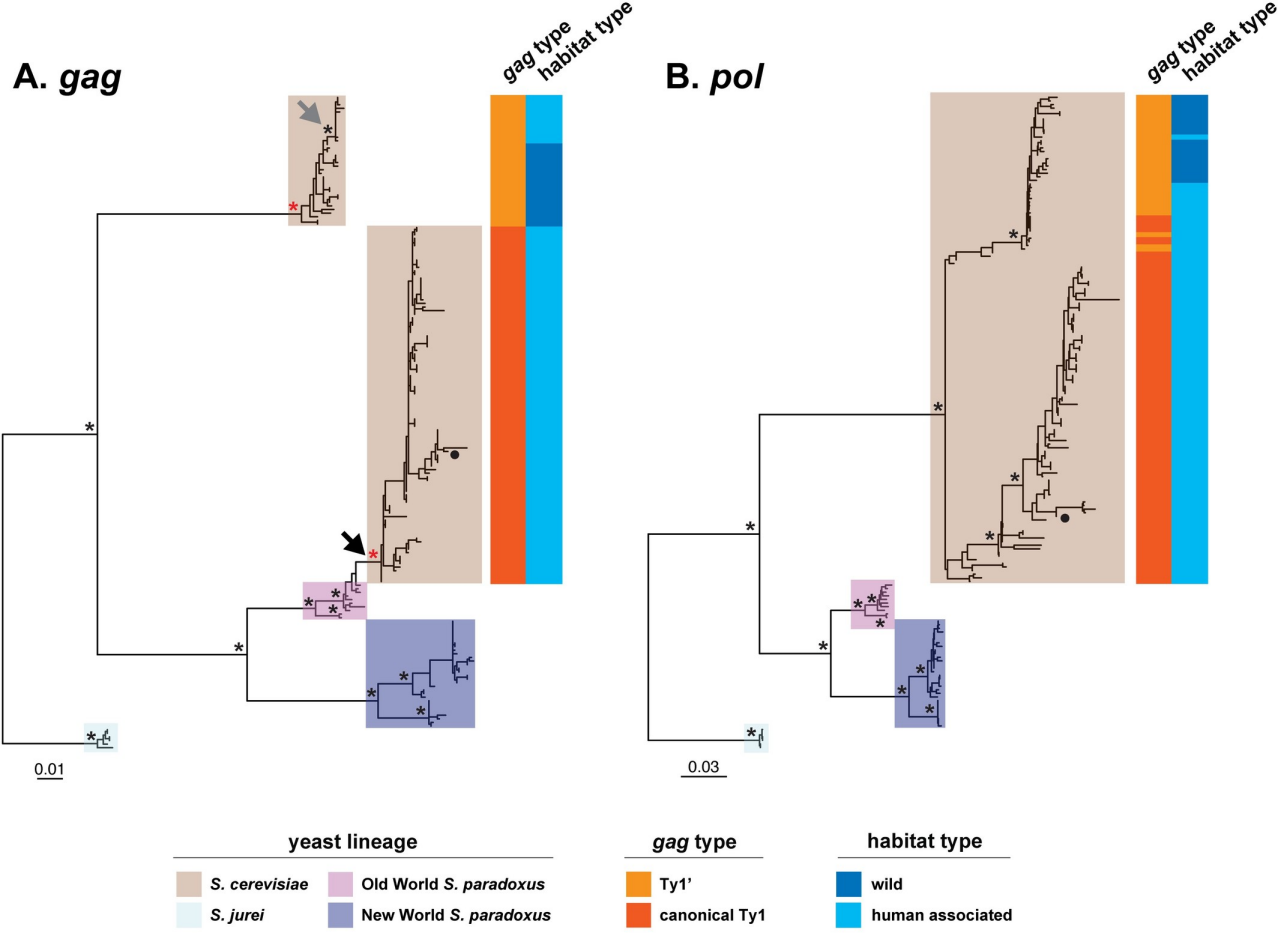
Phylogeny of *gag* and *pol* genes from full-length Ty1 elements in *S*. *cerevisiae* and *S*. *paradoxus*. Maximum likelihood phylogenies of (**A**) *gag* and (**B**) *pol* genes from full-length Ty1 elements in complete PacBio assemblies from 15 strains of *S*. *cerevisiae* and *S*. *paradoxus*, plus two strains of the outgroup species *S*. *jurei*. The scale bar for branch lengths is in units of substitutions per site. Asterisks represent key nodes in the phylogeny with bootstrap support >80%. The filled black circle represents the canonical Ty1-H3 element used in mobility assays. Red asterisks in (**A**) represent nodes for the ancestors of all canonical Ty1 and Ty1’ *gag* sequences, respectively, whose sequences are reconstructed in Fig 5. The black arrow in (**A**) shows evidence that the canonical Ty1 *gag* in *S*. *cerevisiae* is derived from an Old-World *S*. *paradoxus* lineage. The grey arrow in (**A**) shows evidence that elements with Ty1’ *gag* in human-associated *S*. *cerevisiae* have a monophyletic origin derived from a wild *S*. *cerevisiae* ancestor.

Analysis of maximum likelihood phylogenetic trees from this expanded set of strains revealed strikingly discordant histories for Ty1 *gag* (Fig 4A) and *pol* (Fig 4B). Importantly, the phylogenetic history of the *gag* gene is not compatible with the accepted species tree for these taxa [51]. In the *gag* tree, *S*. *cerevisiae* Ty1 sequences are found in two well-supported monophyletic groups (brown background, Fig 4A). One *S*. *cerevisiae gag* clade is the sister group to the ancestor of all *S*. *paradoxus* Ty1 *gag* sequences and contains only elements with Ty1’ type *gag*. The other *S*. *cerevisiae gag* clade–which includes the Ty1-H3 tester element carrying canonical *gag*–is discordantly placed as being derived from the Old World clade of *S*. *paradoxus* Ty1 elements (black arrow, Fig 4A), with the closest affinity to elements from the European lineage of *S*. *paradoxus* represented by CBS432 (S6A Fig). The discordant placement and monophyly of the canonical Ty1 *gag* lineage in *S*. *cerevisiae* are most parsimoniously explained by a single horizontal transfer event from *S*. *paradoxus*. Importantly, all *S*. *cerevisiae* strains isolated from wild sources only contain elements from the Ty1’ clade, despite being sampled from diverse geographic regions around the globe: the Caribbean (UWOPS83-787.3), North America (YPS606, YPS128, SDO2s1), Asia (ZP655.1A), and Europe (ZP568s1) (Fig 4A, S6A Fig). In contrast, human-associated *S*. *cerevisiae* strains have only Ty1 elements with canonical *gag* (DBVPG6044, SK1) or have both types of elements (with canonical Ty1 *gag* or with Ty1’ *gag*) plus their recombinants (S288C, Y12) (Fig 4A, S6 Fig). The Ty1’ *gag* lineage found in human-associated strains is a monophyletic lineage nested within the wild Ty1’ diversity, suggesting a single origin for the introduction of Ty1’ into human-associated strains of *S*. *cerevisiae* (grey arrow, Fig 4A). With the exception of the European *S*. *paradoxus* lineage unexpectedly containing sequences from the *S*. *cerevisiae* canonical Ty1 subfamily, the phylogeny of *S*. *paradoxus* Ty1 *gag* sequences follows the accepted population structure for this species [12]: *S*. *paradoxus* Ty1 elements form an Old World clade comprised of subclades of elements from European (CBS432) and Far Eastern (N-44) lineages, plus a New World clade comprised subclades of elements from S. American (UFRJ50816) and Hawaiian (UWOPS91-917.1) lineages (Fig 4A, S6A Fig).

In contrast to the *gag* phylogeny, the *pol* tree shows the expected species-specific clustering of Ty1 sequences for both *S*. *cerevisiae* and *S*. *paradoxus* (Fig 4B). Within *S*. *cerevisiae*, *pol* sequences form two major groups corresponding generally to the Ty1’ and canonical Ty1 *gag* clades (Fig 4B), but whose coherence and bootstrap support is obscured by recombination in *pol* between the canonical Ty1 and Ty1’ subfamilies (as shown above for the mobility dataset in Fig 2C). The first major *S*. *cerevisiae pol* clade contains all elements that have a Ty1’ *gag*, plus several other recombinant Ty1 elements that have a canonical Ty1 *gag* (e.g. the mosaic Ty1 elements from Y12). The second major *S*. *cerevisiae pol* clade contains elements that have a canonical Ty1 *gag* and includes the Ty1-H3 tester element. Similar to *gag*, all wild strains have a Ty1’ type *pol* and all elements with a canonical Ty1 type *pol* are from human-associated strains. Divergence between the two major *S*. *cerevisiae* groups in *pol* is primarily caused by the regions of *pol* in canonical Ty1 that were acquired by recombination with *S*. *paradoxus* Ty1 (purple region, Fig 3A) or with *S*. *cerevisiae* Ty2 ([8,23]; blue region, Fig 3A), since phylogenetic analysis of a smaller region of *pol* (nucleotides 1700–3000 in Ty1-H3; GenBank: M17806) outside of the regions affected by these recombination events generates a single clade for all *S*. *cerevisiae* strains (S7 Fig, S4 File). As observed for *S*. *paradoxus* Ty1 *gag* sequences, *S*. *paradoxus* Ty1 *pol* sequences cluster by strain according to the accepted global biogeographic relationships for this species [12] (Fig 4B, S6B Fig).

Together, these results suggest that Ty1’ is the ancestral subfamily of Ty1 in *S*. *cerevisiae*, as reflected by its deep divergence from *S*. *paradoxus* in both *gag* and *pol*, and by the unique presence of only pure Ty1’ elements (that have both Ty1’ type *gag* and *pol*) in *S*. *cerevisiae* strains isolated from wild habitats around the world. The conclusion that the Ty1’ subfamily is ancestral in *S*. *cerevisiae* is further supported by the fact that the two truncated Ty1 relics found at high frequency in strains from geographically diverse regions are both from the Ty1’ subfamily (see S4 Fig). These results also suggest that the canonical Ty1 subfamily in *S*. *cerevisiae* is a highly-derived subfamily that acquired a complete *S*. *paradoxus* Ty1 *gag* sequence (as well as parts of *pol* from *S*. *paradoxus* and parts of both LTRs and *pol* from Ty2; purple and blue regions in Fig 3, see also [8,23]) by recombination onto a pre-existing *S*. *cerevisiae* Ty1’-like element, most likely in a human-associated environment. Furthermore, the placement of the *S*. *cerevisiae* canonical Ty1 *gag* clade within the Old World (European) *S*. *paradoxus* clade but sister to the New World *S*. *paradoxus* clade indicates the horizontal transfer involving the *gag* gene event occurred in the Old World, after *S*. *cerevisiae* and *S*. *paradoxus* speciated from one another and divergence of the major worldwide *S*. *paradoxus* lineages had occurred.

### Divergence between canonical Ty1 and Ty1’ Gag occurs outside functionally-characterized residues

**Fig 5:**
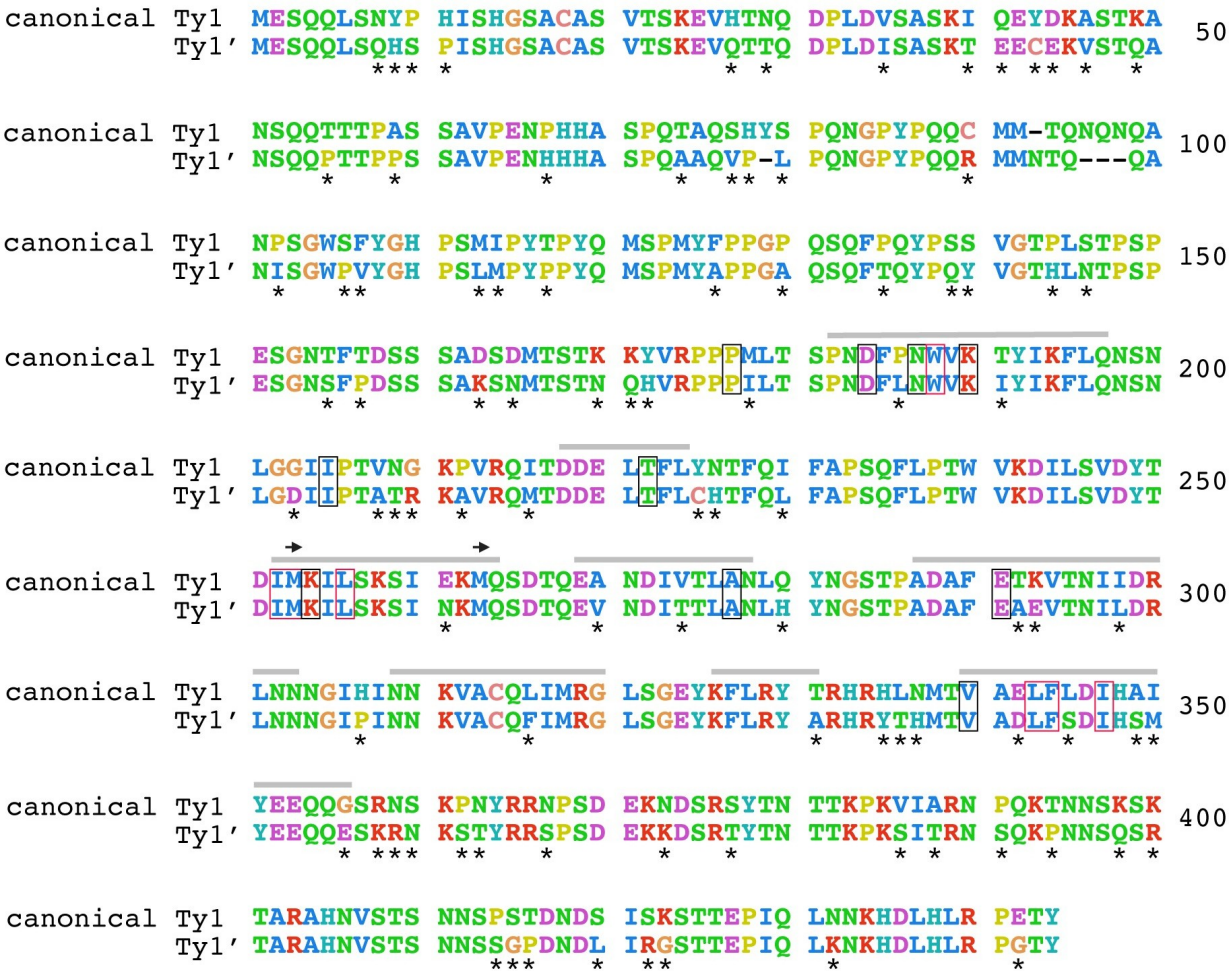
Amino acid divergence between ancestral canonical Ty1 and ancestral Ty1’ *gag* sequences. Codon-based alignment of maximum likelihood reconstructed ancestral states of all *S*. *cerevisiae* canonical Ty1 and all Ty1’ *gag* sequences, respectively, excluding two elements that are recombinant in *gag* (Y12_f109 and S288c_f486). Amino acids with similar properties were colorized as follows: KR (positively charged) = red; AFILMVW (hydrophobic side chains) = blue; NQST (polar uncharged side chains) = green; HY = teal; C = salmon; DE (negatively charged) = purple; P = yellow; G = orange. Amino acid substitutions between ancestral sequences are denoted by asterisks. Amino acids sites in Ty1 Gag with known function [32,35] are annotated as follows: sites that cause resistance to p22-based CNC are shown in black boxes; sites that are important for Ty1 protein maturation are shown in red boxes; grey bars represent predicted helical regions; and alternative start codons for p22 are shown as arrows.

Comparison of Ty1 genomic content with mobility phenotypes above revealed that strains encoding only Ty1’ *gag* cannot strongly repress mobility of a Ty1-H3 tester element (Table 1). These mobility assays imply that sequence divergence between canonical Ty1 and Ty1’ in Gag may affect the ability of Ty1’ elements to confer CNC on a canonical Ty1 element. To understand how molecular evolution along the branch separating the canonical Ty1 and Ty1’ subfamilies relates to potential functional divergence in *gag*, we reconstructed ancestral *gag* sequences for all canonical Ty1 elements and Ty1’ elements, respectively. Codon-based alignment of ancestral canonical Ty1 and Ty1’ *gag* sequences revealed 93 amino acid and three insertion/deletion substitutions across Gag, 40 of which are in the p22 region (Fig 5). Consistent with the extensive divergence in *gag* occurring along functional Ty1 lineages evolving in distinct species rather than rapid sequence evolution within species due to positive selection, we found that purifying selection was the prevailing mode of molecular evolution across both the entire *gag* gene (dN = 0.125; dS = 0.252; dN/dS = 0.496) and the p22 region (dN = 0.128; dS = 0.289; dN/dS = 0.443) (see also Kim *et al*. [7]). Moreover, the two alternative start codons for p22 are conserved between canonical Ty1 and Ty1’ ancestors [32,34], as are the ten CNC^R^ residues shown to provide resistance to p22 [35], the seven amino acids shown to be important for Ty1 protein maturation [35], and the seven nucleotides required for +1 frameshifting to create the Gag-Pol fusion protein [52]. Amino acid substitutions differentiating canonical Ty1 and Ty1’ occur at similar proportions in the p22 region versus the non-p22 portions of Gag (*P* = 0.907; Fisher’s Exact Test) as well as in the nine predicted helical regions relative to remaining non-helical regions of Gag (*P* = 0.123; Fisher’s Exact Test). Similarly, nucleotide substitutions between canonical Ty1 and Ty1’ occur at similar proportions in the regions of *gag* that produce anti-sense RNAs [53] relative to regions that do not produce anti-sense RNAs (*P* = 0.745; Fisher’s Exact Test). These results suggest that the Ty1’ subfamily has the capacity to code for a p22-like molecule and that potential functional divergence between canonical Ty1 and Ty1’ Gag occurs outside residues currently known to affect Ty1 protein function, maturation or resistance to p22.

## Discussion

Here we combine *in vivo* transposition assays with high-resolution phylogenomics to show that variation in the ability to repress mobility of a Ty1 element carrying a canonical *gag* in *S*. *cerevisiae* is a consequence of horizontal transfer of a *gag* gene from a closely related yeast species, *S*. *paradoxus*. Additionally, our results indicate that canonical Ty1 CNC is likely to be widespread in both species and vary in strength as a function of the genomic content of specific Ty1 subfamilies. The correlation of the presence of canonical Ty1 *gag* in full-length elements with the restrictive Ty1-H3 mobility phenotype–demonstrated most clearly by the restrictive Y12 strain–also provides compelling and independent evolutionary genomic evidence to support functional studies showing that canonical Ty1 CNC is mediated by sequences within the Ty1 *gag* region [30,32].

Our work reveals that, while the presence of the Ty1 family is ancestral to both species, the ability of *S*. *cerevisiae* strains to strongly repress Ty1-H3 mobility was likely acquired after speciation through horizontal transfer. Acquisition of a *S*. *paradoxus gag* gene by *S*. *cerevisiae* canonical Ty1 explains the surprising similarity between *S*. *cerevisiae* and *S*. *paradoxus* Ty1 *gag* sequences first reported here, as well as the ability of *S*. *paradoxus* strains with full-length Ty1 elements to restrict mobility of a heterospecific *S*. *cerevisiae* Ty1 tester element shown here and in previous studies [30,37]. Our observation of restrictive strains in *S*. *paradoxus* with full-length Ty1 elements from both European and Far Eastern lineages (Table 1) suggests that canonical Ty1 CNC was present in the ancestor of all Old World *S*. *paradoxus* lineages. However, Ty1 mobility assays in American and Hawaiian *S*. *paradoxus* strains with full-length Ty1 elements are needed to establish if canonical Ty1 CNC is also present in New World *S*. *paradoxus* lineages, which would suggest it was present in the common ancestor of all *S*. *paradoxus* lineages prior to colonizing worldwide habitats. Further studies on Ty1 from other species in the *Saccharomyces* genus and related Ty elements are also needed to understand where and when canonical Ty1 CNC first evolved. Previous work indicates that *S*. *cerevisiae* Ty2 is not under a self-encoded form of CNC nor is Ty2 responsive to p22 based Ty1 CNC [35]. These results suggest that Ty1 CNC could have evolved after the divergence of Ty1 and Ty2, however the complex history of the Ty1/Ty2 superfamily in the *Saccharomyces* genus [13,22,54] currently leaves open many other possibilities as to when canonical Ty1 CNC ultimately originated.

Our findings raise a number of intriguing questions about the p22-based mechanism of Ty1 CNC that can be explored in future studies. The inability of *S*. *cerevisiae* strains containing only Ty1’ *gag* to strongly repress Ty1-H3 mobility implies potential functional divergence in the *gag* genes of the canonical and Ty1’ subfamilies. While sequence analysis suggests Ty1’ is potentially capable of manifesting p22-based CNC (Fig 5), further work is needed to address whether this is indeed the case. If it can be shown that Ty1’ is capable of producing a functional p22 that exerts CNC on other Ty1’ elements, it will be important to evaluate which sequences in *gag*/p22 outside of currently functionally-characterized residues are responsible for this potential functional divergence. If Ty1’ does exert CNC *via* a p22-based mechanism, it is also possible that the different truncated Ty1’ relics found in multiple *S*. *cerevisiae* strains [14] may represent domesticated Ty1 restriction factors that repress Ty1’ mobility, similar to domesticated *gag* genes that inhibit murine or sheep retrovirus replication [36,55,56]. Potential functional divergence in the ability of Ty1’ to repress canonical Ty1 also raises questions about possible regulatory interactions among these two subfamilies. At this point, it is clear that strains containing elements with both canonical Ty1 and Ty1’ *gag* (e.g. native S288c, native Y12, and populated YPS606) restrict Ty1-H3, and thus Ty1’ elements do not dominantly inhibit CNC mediated by canonical Ty1 p22. If one assumes similar levels of Ty1*his3-AI* expression and RNA splicing in different strains, there is also some indication that permissive strains containing full-length Ty1’ elements (UWOPS83-787.3 and YPS606) have lower Ty1-H3 mobility than permissive strains that lack any full-length Ty1 elements (UWOPS05-227.2 and L-1374). This observation raises the possibility that Ty1’ can limit Ty1-H3 mobility to some degree through a CNC-like mechanism. Additionally, many questions remain about the basis of quantitative variation in Ty1-H3 mobility across strains, including why Ty1-H3 mobility is similar for genomes that vary two-fold in the number of canonical Ty1 elements (S288c and DBVP6044). The expression level of individual Ty1 insertions has previously been shown to differ substantially as a function of genomic location [57], and thus the genomic context of Ty1 insertions (in addition to overall copy number) likely contributes to variation in Ty1-H3 mobility among strains. To address these questions, future functional studies on Ty1 CNC in *S*. *cerevisiae* will require development and application of robust genetic techniques to characterize the mobility and CNC competence of a wider range of native Ty1 elements (including members of the Ty1’ subfamily) in a diverse panel of natural isolates and isogenic strains derived from natural strains populated with various Ty1 elements.

Results presented here extend our understanding of the evolution of Ty1 in *S*. *cerevisiae* and *S*. *paradoxus*. Combined with previous work by Jordan and McDonald [8,23], our results suggest that the Ty1-H3 element used in most studies on Ty1 expression or function is a highly-derived element that acquired sequences from both *S*. *paradoxus* Ty1 and *S*. *cerevisiae* Ty2, most likely in a human-associated environment. How and when these events happened remain to be determined, although the importance of homologous recombination in both events is clear. Decoding the history of the canonical Ty1 subfamily will need to explain the somewhat paradoxical observation that this subfamily confers strong repression against itself but also apparently has high fitness, as reflected by its high copy number in strains that carry this subfamily. Understanding the history of these events may be challenging since the lack of overlap in the sequences acquired from *S*. *paradoxus* and Ty2 prevents their relative ordering, and the ongoing effects of recombination between canonical Ty1 and Ty1’ in strains that carry both subfamilies may obscure efforts to reconstruct the original sequences involved. Regardless, the highly-derived nature of the Ty1 element should caution against naively interpreting the structure, function or evolution of transposable elements isolated in domesticated lab strains as being reflective of the natural genome biology in yeast or other species.

Our results also provide evidence that horizontal transfer events may not always lead to the transfer of complete transposable element sequences, and that recombination after horizontal transfer may lead to the evolution of novel hybrid transposable elements. The presence of *S*. *paradoxus* sequences in canonical Ty1 in both the *gag* (red, Fig 3A) and *pol* (purple, Fig 3A) regions could either reflect independent recombination events of horizontally transferred Ty1 sequences from *S*. *paradoxus* or indicate that a larger fragment of *S*. *paradoxus* was originally transferred onto the ancestor of canonical Ty1 (with the sequence between *gag* and *pol* subsequently converted back to a Ty1’-like state by recombination). Given the relatively common occurrence of introgression of nuclear genes from *S*. *paradoxus* into *S*. *cerevisiae* [20,58–62], multiple horizontal transfer or recombination events may have occurred in the Old World to create the canonical Ty1 lineage, and additional horizontal transfer events of Ty1 are yet to be discovered in other geographic areas where these two species coexist in nature. Horizontal transfer of Ty1 sequences from *S*. *paradoxus* into *S*. *cerevisiae* may have occurred by transmission of a Ty1 VLP or RNA during mating between *S*. *paradoxus* and *S*. *cerevisiae*. Alternatively, *S*. *paradoxus* Ty1 sequences may have entered *S*. *cerevisiae* by introgression of a segment of the nuclear genome carrying one or more *S*. *paradoxus* Ty1 elements.

Finally, the evolution of Ty1 in *S*. *cerevisiae* described here may inform aspects of the history of domestication and biogeography of this species. Recent work proposed that *S*. *cerevisiae* beer strains have genetic contributions from European and Asian lineages [63]. The acquisition by canonical Ty1 of sequences related to both European *S*. *paradoxus* Ty1 and Ty2 (which is proposed to have arisen in *S*. *cerevisiae* by horizontal transfer from the Asian species *S*. *mikatae* [13,22]) in human-associated strains supports this model. Likewise, the inference that Ty1’ is the ancestral lineage in *S*. *cerevisiae* and that wild strains of *S*. *cerevisiae* lack canonical Ty1 suggests that Ty1’ may be a useful marker for studying biogeography in this species. For example, if the “out-of-China” origin proposed for the ancestral range of wild *S*. *cerevisiae* is correct [20,64], our findings predict that that only the Ty1’ subfamily should be found in ancestral woodland strains of *S*. *cerevisiae* from China. Answers to these and other questions should be facilitated by advances in the assembly of noisy long-read datasets [16], and development of computational techniques to resolve the presence of canonical Ty1 and Ty1’ sequences from abundant short-read datasets for *S*. *cerevisiae* [20,49,60,61,63,65–72].

## Materials and methods

### Strains, plasmids, and genetic techniques

All strains used in this study are listed in S5 File. Strains used for Southern analysis, mobility assays and the majority of genome sequencing experiments (S288c, Y12, DBVPG6044, UWOPS83-787.3, YPS606, UWOPS05-227.2, L-1374, CBS432, N-44 and YPS138) are *MATα* haploid derivatives of *S*. *cerevisiae* and *S*. *paradoxus* strains from the SGRP [44]. Additional monosporic wild-type *S*. *cerevisiae* strains used for genome sequencing (SDO2s1, ZP568s1, ZP655.1A) were reported previously [49]. For the *S*. *cerevisiae* SGRP strains used in mobility experiments, the *HIS3* universal gene blaster pBDG652 digested with EcoRI and SphI was used to generate *his3-Δ200hisG* deletion alleles in SGRP strains [30,73]. For the *S*. *paradoxus* strains used in mobility experiments, *KanMX* was replaced by *NatMX* by homologous recombination as previously described [74]. For *S*. *cerevisiae* strains YPS606, UWOPS05-227.2 and L-1374, *his3-Δ200hisG* deletion strains were subsequently “populated” with canonical Ty1 elements following galactose-induced expression of pGTy1-H3 [45], and copy number estimates were determined by Southern analysis as described previously [30]. To generate strains for determining the level of Ty1 mobility, native or populated haploid *MATα his3-Δ200hisG S*. *cerevisiae* strains were transformed with a Ty1*his3-AI URA3* centromere plasmid pOY1 (pBDG633) [75]. Likewise, native haploid *MATα NatMX S*. *paradoxus* strains were transformed with a Ty1*neo-AI* plasmid (pBDG954) constructed by subcloning a BstE II–Eag I fragment from pGTy1-H3PtefKAN-AI [41] into pBDG633. Restriction endonucleases, Phusion DNA polymerase, and T4 DNA ligase were purchased from New England Biolabs (Ipswich MA). Plasmids were verified by restriction analysis and DNA sequencing. Standard yeast genetic and microbiological procedures were used in this work, including media preparation and DNA transformation [76–78].

### Southern blot hybridization

A single colony from each strain was inoculated in 10 ml of YEPD medium and grown to saturation at 30°C, and total DNA was isolated as described previously [45]. Approximately 10 μg of DNA was digested with BglII and resolved on a 0.6% agarose gel for 16 hours at 30V. DNA fragments were transferred via capillary action to Hybond-N membrane (GE Healthcare, Aurora OH) and UV-crosslinked according to the supplier’s specifications (Spectroline, Westbury NY). ^32^P-labeled DNA probes containing *gag* sequences were made by randomly primed DNA synthesis with Amersham Megaprime DNA labeling System (GE Healthcare). The *gag* sequences were amplified by PCR from plasmids pGTy1-H3 (GenBank: M18706; nucleotides 335–1496) and pGTy2-917 (GenBank: KT203716; nucleotides 333–1490) [79]. DNA for the Ty1 probe was amplified with primers 5’-TGGTAGCGCCTGTGCTTCGGTTAC-3' and 5'-CATGTTTCCTCGAGTTAGTGAGCCCTGGCTGTTTCG-3' and Phusion DNA polymerase (New England Biolabs). DNA for the Ty2 probe was generated with primers 5’-TGGTAGCGCCTATGCTTCGGTTAC-3’ and 5’-GCAATATTGTGAGCTTTTGCTGCTCTTGG-3’. Hybridization was performed at 68°C overnight in a buffer containing buffer containing 6X SSC, 5X Denhardt’s solution, 0.5% SDS, and 100 μg/μl single-strand salmon sperm DNA. Blots were washed at 68°C, twice with 2X SSC + 0.1% SDS for 30 min, followed by two washes with 1X SSC + 0.1% SDS for 15 min. Membranes were exposed to storage phosphor screens followed by scanning with Molecular Dynamics Storm PhosphorImager (GE Healthcare).

### Ty1 mobility

The frequency of Ty1*his3-AI* and Ty1*neo-AI* mobility was determined as described previously with minor modifications [30,39,41]. Briefly, a single colony from a SC-Ura plate incubated at 30°C was resuspended in 1 ml of water and 5 μl of cells was added to quadruplicate one-ml cultures of SC-Ura liquid medium. The cultures were grown for 3 days at 22°C, washed, diluted, and spread onto SC-Ura, and SC-His-Ura (for Ty1*his3-AI*) or YEPD + Geneticin (G418; 200 μg/ml for Ty1*neo-AI*) (ThermoFisher, Waltham MA) plates. To minimize flocculence, 0.25 M NaCl was used for cell washes and dilutions prior to plating [80]. The frequency of Ty1 mobility is defined as the number His^+^ Ura^+^ or G418^R^ colonies divided by the number of Ura^+^ colonies per ml of culture. Standard deviations were calculated from the number Ty1 mobility events detected per 1 ml culture. To determine the fraction of colonies on YEPD +/- Geneticin that retain pBDG954, we analyzed strain YPS138 (DG3912, Table 1, Sup File 5). Over 95% of the mobility events obtained on YEPD + G418 remain Ura^+^ (86/90), and over 98% (377/382) retain pBDG954 after growth in SC-Ura liquid medium. Thus, the same events are revealed in Ty1 mobility assays using *his3-AI or neo-AI* indicator genes and slightly different plating schemes, because pBDG954 retention remains very high on YEPD +/- G418 following growth in SC-Ura.

### Isolation of genomic DNA for PacBio sequencing

Genomic DNA was extracted using the Wizard Genomic DNA purification kit (Promega, Madison WI) according to the manufacturer’s instructions for yeast with minor modifications (https://dx.doi.org/10.17504/protocols.io.rved63e). A single colony of each strain was inoculated in 7 ml of YPD media and cultured for 20–24 hours at 30°C. Approximately 6 ml of culture for each strain was transferred to 4, 1.5 ml tubes and centrifuged at 16,162 relative centrifugal force (RCF) for 2 min at room temperature. The resulting cell pellet was resuspended in 293 μl of 50mM EDTA, pH 8.0 and incubated with 100 units of Lyticase (Sigma-Aldrich, Saint Louis MO) at 37°C for 21 hours. The resulting spheroplasts were centrifuged at 16,162 RCF for 2 min, resuspended in 300 μl of Promega Nuclei Lysis Solution, followed by adding 100 μl of Promega Protein Precipitation Solution, and incubated on ice for 5 min. Lysates were centrifuged at 16,162 RCF for 10 min. The supernatant was transferred to a 1.5 ml tube containing 300 μl of isopropanol and tubes were inverted 50 times to facilitate DNA precipitation. DNA was pelleted by centrifugation at 16,162 RCF for 10 min. The DNA pellet was washed with 300 μl of 70% ethanol, centrifuged at 16,162 RCF for 5 min and air dried for 15 min. The DNA pellet was resuspended in 50 μl of Promega DNA rehydration solution by gentle pipetting. Samples were treated with 5.25 units of RNAse A (Qiagen, Hilden Germany) at 37°C for 1 hour. Samples were incubated at 65°C for 45 min and stored at 4°C. DNA was centrifuged at 16,162 RCF for 10 min and supernatants of the four extracts were pooled, and additional purification steps were performed as follows. Qiagen DNA Hydration solution was added to bring the final volume from ~180 μl to 200 μl/strain. Three microliters of Qiagen RNase A Solution was added to each tube followed by incubation at 37°C for 1 hour. The sample was transferred on ice and 0.5 volume of Qiagen Protein Precipitation Solution was added, followed by 2 volumes of cold 100% ethanol. Tubes were mixed by inversion 50 times and incubated on ice for 15 min. Precipitated DNA was pelleted at 16,162 RCF for 10 min and washed with 70% ethanol followed by short spin 16,162 RCF for 3 min. The supernatant was removed, and the DNA pellet was air dried for 15 min. The DNA pellet was resuspended in 110 μl of Qiagen DNA hydration Solution by gentle pipetting, followed by incubation at 37°C for 1.5 hours to fully dissolve the DNA. An additional spin at 16,162 RCF for 10 min was performed in order to pellet any impurities. Supernatants were transferred to fresh tubes and stored at 4°C or -20°C prior to making PacBio libraries.

### Genome sequencing and assembly

PacBio sequences were generated for *S*. *cerevisiae* using the PacBio RS II platform [81]. For *S*. *cerevisiae* genomes, total DNA was purified with 1x cleaned AMPure beads (Beckman Coulter, Pasadena CA) and the quantity and quality were assessed using Nanodrop and Qubit assays. Five micrograms of purified DNA samples were sheared to approximately 20 Kb using gTubes (Covaris, Woburn MA) at 1000 x g. Sheared DNA was recovered by purification with 1:1 vol ratio of AMPure beads. Sheared DNA was treated with Exonuclease V11 (New England Biolabs), at 37°C for 15 min. The ends of the DNA were repaired by first incubating for 20 min at 37°C with damage repair mix supplied in the SMRTbell library kit (Pacific Biosciences, Menlo Park CA). This was followed by a 5-minute incubation at 25°C with end repair mix in the SMRTbell library kit. End-repaired DNA was then cleaned using 1:1 volume ratio of AMPure beads and 70% ethanol washes. End-repaired DNA was ligated to SMRTbell adapter overnight at 25°C. Ligation was terminated by incubation at 65°C for 10 min followed by exonuclease treatment for 1 hour at 37°C. The SMRTbell library was purified with 1:1 volume ratio of AMPure beads. The quantity of library and therefore the recovery was determined by Qubit assay and the average fragment size determined by Fragment Analyzer. Size-selection was performed on Sage Blue Pippin Prep using 0.75% agarose cassette and S1 marker. The size-selected SMRT bell was recovered using 1:1 volume ratio of AMPure beads and quantified by Qubit. The final average size was between 17–23 Kb. SMRTbell libraries were annealed to sequencing primer at values predetermined by the Binding Calculator (Pacific Biosciences) and a complex made with the DNA Polymerase (P6/C4 chemistry). The complexes were bound to Magbeads and this was used to set up the required number of SMRT cells for each sample. SMRT sequencing was performed using the PacBio RS II system with a movie time 360 min. Genome assembly was performed using the RS_HGAP_Assembly.3 protocol in the SMRT Analysis package version 2.3.0. Assembly statistics and quality were determined using Quast (version 4.2) [82] and Mummerplot (version 3.23) [83] relative to the UCSC sacCer3 reference assembly. Raw PacBio reads and assemblies were submitted to ENA under accession PRJEB33725.

### Annotation of Ty elements

HGAP assemblies for the genomes reported here plus a complementary set of HGAP assemblies from *S*. *cerevisiae*, *S*. *paradoxus*, and the outgroup species *S*. *jurei* [18,43] were used to identify Ty elements using a modified strategy similar to Carr *et al*. [13]. RepeatMasker (version 4.0.5; options: -e wublast -s -xsmall -nolow -no_is) (http://repeatmasker.org) was used to find all Ty fragments with similarity to a custom database of canonical Ty sequences derived from those reported in Carr *et al*. [13] that was updated to fix several small errors and include a version of the Tsu4 element from *S*. *paradoxus* [19] (S6 File). Inspection of raw RepeatMasker results identified a number of false positive matches to divergent sequences, LTR fragments from full-length or truncated elements were labeled as the incorrect family, tandem duplications of Ty elements that share a common LTR sequence that were incorrectly joined, and fusion of nearby solo LTRs fragments to full or truncated Ty elements. To fix these errors, we applied a series of automated filtering and editing operations to the raw RepeatMasker .out files: (i) false positive Ty predictions were filtered out of the RepeatMasker .out file by removing all matches to fragments with >20% divergence to the canonical Ty internal or LTR sequence; (ii) LTR fragments from full-length Ty1 and Ty2 elements were modified to match the name of the internal region found in contiguous clusters of Ty fragments with the same RepeatMasker id; (iii) tandemly-arrayed Ty elements that share an internal LTR sequence were split into distinct copies with shared LTR sequences being represented in each component copy; and (iv) flanking solo LTRs that were incorrectly joined to a complete or truncated element were split into distinct, non-overlapping copies. The final modified RepeatMasker .out file was converted to BED12 format with all fragments having the same RepeatMasker ID joined into a single BED12 record. Each Ty element in the BED12 file was categorized structurally as full-length (f, internal region present and total length >95% of canonical length), truncated (t, internal region present and total length <95% of canonical length), or solo LTRs (s, LTR present but no match to internal region). BED12 files for each strain (S7 File) were then used to summarize counts of all Ty element structural classes. Following Yue *et al*. [18], counts of solo LTRs for Ty1 and Ty2 were pooled because of the similarity of their LTR sequences.

### Alignment and sequence analysis of Ty1 elements

BED12 files were used to extract fasta sequences oriented relative to the positive strand of each full-length Ty element using BEDtools getfasta (version 2.26.0; options: -name -s) [84]. Fasta files of full-length Ty1 sequences from all strains were concatenated together with the Ty1-H3 and Ty2 canonical elements and aligned using mafft (version 7.273) [85] (S8 File). *gag* and *pol* regions were annotated based on Ty1-H3 coordinates in the resulting multiple alignment. For sliding window analysis, a subset of aligned fasta sequences was extracted and gap-only sites were removed using Seaview (version 4.7) [86]. Sequence divergence between pairs of Ty1 elements was estimated using Kimura’s 2-parameter substitution model was calculated for 50 bp windows with a 10 bp step size in R (version 3.5.2) using the spider (version 1.5) and phangorn (version 2.4) packages [87,88]. For clustering analysis, complete sequence and region-specific alignments (for *gag* and *pol*) from the subset of strains with mobility data plus Ty1-H3 were extracted and gap-only sites were removed using Seaview (version 4.7) [86]. Aligned sequences were then clustered using the BIONJ algorithm with a Kimura 2-parameter substitution model in Seaview (version 4.7) [86,89] and resulting trees were visualized in R (version 3.5.2) using the APE package (version 5.2) [90]. Phylogenetic networks of sequences from the mobility dataset plus Ty1-H3 were generated using uncorrected P-distance and the Neighbor Net algorithm in SplitsTree 4.15.1 [91,92]. For maximum-likelihood phylogenetic analysis, region-specific alignments for the expanded dataset plus Ty1-H3 (excluding one recombinant element each from S288c and Y12) was performed using raxmlHPC-PTHREADS-AVX (version 8.2.4; options -T 28 -x 12345 -p 12345 -b 12345 -N 100 -m GTRGAMMA) [93]. Resulting phylogenetic trees were visualized in FigTree 1.4.4. Ancestral states for *gag* nucleotide sequences in the maximum likelihood tree were reconstructed using raxmlHPC-PTHREADS-AVX (version 8.2.4; options -T 28 -f A -m GTRGAMMA) [93]. Fasta sequences for the ancestors of all canonical Ty1 and Ty1’ *gag* sequences, respectively, were extracted and re-aligned using PRANK (v.170427; options: -codon -F) [94]. Codon-aligned ancestral *gag* sequences were used to estimate dN, dS, and dN/dS ratios under PAML model M0 using ETE3 [95,96]. Codon-based nucleotide alignments were converted to amino acids and visualized in Seaview (version 4.7) [86]. Regions of Ty1 retained in truncated elements were determined by aligning individual truncated elements to the Ty1-H3 reference sequence using minimap2 (version 2.17: options -x spliced -a) [97], converting to BAM format with SAMtools (version 1.8) [98], projecting aligned fragments onto the Ty1-H3 reference sequence using BEDtools bamtobed (version 2.28.0; options: -splitD) [84], and visualizing using ggplot2 [99].

## Supporting information

S1 Fig Southern blots of canonical Ty1-H3 *gag* hybridized with total DNA from *Saccharomyces* Genome Resequencing Project (SGRP) strains. (**A**) Schematic of the Ty1 element, showing the *gag* and *pol* open reading frames (rectangles) and LTRs (arrowheads). The radiolabeled probe used for Southern blots is obtained from the *gag* gene (underlined). The location of the BglII restriction site within Ty1-H3 *pol* is shown above the schematic. The complete Ty1-H3 element is 5918 bp in length and *gag* probe is 1,162 bp in length. Southern blot results for (**B**) *S*. *cerevisiae* and (**C**) *S*. *paradoxus*. Dots in panels (B) and (C) represent strains that were selected for Ty1 mobility assays.
(TIF)

S2 Fig Sequence divergence between Ty1’ and elements with recombination between canonical Ty1 and Ty1’ in the *gag*. Sliding window analysis of pairwise sequence divergence between (**A**) Y12_f109 vs. Ty1’ and (**B**) S288c_f486 vs. Ty1’. The pure Ty1’ element Y12_f208 is used in both panels. Coordinates shown are relative to the multiple sequence alignment and are therefore the same for all panels. Divergence measured in substitutions per site was calculated using a Kimura 2-parameter model in overlapping 50 bp windows with a 10 bp step size.
(TIF)

S3 Fig Phylogenetic networks Ty1 sequences from *S*. *cerevisiae* strains with Ty1-H3 mobility phenotypes. Neighbor-Net phylogenetic networks using uncorrected P-distances were constructed using (**A**) complete sequences, (**B**) *gag* sequences, or (**C**) *pol* sequences from full-length Ty1 elements. Nodes where incompatible partitions of sequence variation (“splits”) occur in the data because of recombination between canonical Ty1 and Ty1’ are connected by multiple edges. Bands of parallel edges would collapse to individual branches if no conflicting splits due to recombination existed in the data. Recombinant elements between Ty1’ and canonical Ty1 within *gag* are starred (*Y12_f109; **: S288c_f486).
(TIF)

S4 Fig Truncated Ty1 elements in *Saccharomyces* strains with Ty1-H3 mobility phenotypes. Schematic representation of regions of canonical Ty1-H3 element retained in individual truncated elements in strains with Ty1-H3 mobility data. Truncated elements are defined as having some non-LTR internal region of Ty1 present but have a total length that is <95% of the canonical Ty1 element. Strains with full-length elements are labelled in the same colors as in Fig 2. Fragments of the same truncated element are connected by dashed lines. Truncated elements labelled as Ty1’ relics were previously reported by Bleykastens-Grosshans *et al*. [14].
(TIF)

S5 Fig Sequence divergence between subfamilies of Ty1 and Ty2. Sliding window analysis of pairwise sequence divergence between (**A**) canonical Ty1 vs. Ty2, and (**B**) Ty1’ vs. Ty2. Similarity between a subset of Ty1 elements and Ty2 in parts of the LTRs *pol* was previously reported in [8,23] and proposed to have arisen by recombination between these Ty families. Recombination between Ty1 and Ty2 must have occurred on an ancestor of the canonical Ty1 subfamily since high divergence between canonical Ty1 and Ty1’ in the LTRs and 3’ region of *pol* (blue, Fig 3A) spans the same regions that have high similarity between canonical Ty1 and Ty2 (blue, S5A Fig) but have high divergence between Ty1’ and Ty2 (S5B Fig). Identifiers for elements shown are: RepBase TY2#LTR/Copia (Ty2), DBVPG6044_f486 (pure canonical Ty1); Y12_f208 (pure Ty1’). Divergence measured in substitutions per site was calculated using a Kimura 2-parameter model in overlapping 50 bp windows with a 10 bp step size.
(TIF)

S6 Fig Strain-labelled phylogeny of *gag* and *pol* genes from full-length Ty1 elements in *S*. *cerevisiae* and *S*. *paradoxus*. Maximum likelihood phylogenies of (**A**) *gag* and (**B**) *pol* genes from full-length Ty1 elements in complete PacBio assemblies from 15 strains of *S*. *cerevisiae* and *S*. *paradoxus*, plus two strains of the outgroup species *S*. *jurei*. The scale bar for branch lengths is in units of substitutions per site. Nodes labelled by asterisks in Fig 4 are shown with bootstrap support values. Ty1 element identifiers are a composite of strain name followed by a strain-specific unique numerical identifier prefixed by “f” indicating that it is a full-length element. Tree files in Newick format for *gag* and *pol* can be found in S2 File and S3 File, respectively. Aligned fasta sequences for all full-length Ty1 elements can be found in S8 File.
(TIF)

S7 Fig Strain-labelled phylogeny of non-recombinant *pol* region from full-length Ty1 elements in *S*. *cerevisiae* and *S*. *paradoxus*. Maximum likelihood phylogenies of region of *pol* gene outside regions of recombination (nucleotides 1700–3000 in M18706) between canonical Ty1 and either *S*. *paradoxus* Ty1 or Ty2 from full-length Ty1 elements in complete PacBio assemblies from 15 strains of *S*. *cerevisiae* and *S*. *paradoxus*, plus two strains of the outgroup species *S*. *jurei*. The scale bar for branch lengths is in units of substitutions per site. Bootstrap support is shown for nodes with values >95%. Ty1 element identifiers are a composite of strain name followed by a strain-specific unique numerical identifier prefixed by “f” indicating that it is a full-length element. Tree file in Newick format for the non-recombinant region of *pol* can be found in S4 File. Aligned fasta sequences for all full-length Ty1 elements can be found in S8 File.
(TIF)

S1 File Assembly statistics and Ty content in PacBio assemblies of *Saccharomyces* species. Strains from the current work are labeled Czaja; strains from published assemblies are labeled by the last name of the first author of the respective paper [18,42,43]. Counts of Ty elements are based on structural classification of fragments from the same RepeatMasker annotation: full-length (internal region present and total length >95% of canonical length), truncated (internal region present and total length <95% of canonical length), or solo LTRs (LTR present but no match to internal region). Following Yue *et al*. [18], counts of solo LTRs for Ty1 and Ty2 were pooled because of the similarity of their LTR sequences.
(TXT)

S2 File Maximum likelihood tree for the complete Ty1 *gag* region. Newick-formatted maximum likelihood tree file based on *gag* sequences of full-length Ty1 elements in the expanded dataset plus the canonical Ty1-H3 element (M18706). Node labels represent bootstrap support based on 100 replicates and branch lengths are in substitutions per site.
(TXT)

S3 File Maximum likelihood tree for the complete Ty1 *pol* region. Newick-formatted maximum likelihood tree file based on *pol* sequences of full-length Ty1 elements in the expanded dataset plus the canonical Ty1-H3 element (M18706). Node labels represent bootstrap support based on 100 replicates and branch lengths are in substitutions per site.
(TXT)

S4 File Maximum likelihood tree for the non-recombinant region of Ty1 *pol*. Newick-formatted maximum likelihood tree file based on *pol* sequences corresponding to nucleotides 1700–3000 of M18706 of full-length Ty1 elements in the expanded dataset plus the canonical Ty1-H3 element (M18706). Node labels represent bootstrap support based on 100 replicates and branch lengths are in substitutions per site.
(TXT)

S5 File List of *Saccharomyces* strains used in this study. Columns provide information for the species, strain identifier, genotype, parental strain, geographic origin, source, purpose in the current study, and the original reference for each strain. Identifiers for SGRP strains from Cubillos *et al*. [44] are from the National Collection of Yeast Cultures and Yue *et al*. [18] are from the Liti lab collection. Metadata for the geographic origin and source of SGRP strains is from Liti *et al*. [12] and Bergstrom *et al*. [66]. Identifiers for strains reported in this study are from the Garfinkel lab collection. The *his3-Δ200hisG* deletion was introduced using plasmid pBDG652 as described in Garfinkel *et al*. [30]. The plasmid pOY1 (pBDG633: Ty1*his3-AI URA3 CEN*) was reported in Lee et *al*. 1998 [75], and the plasmid pBDG954 (Ty1*neo-AI URA3 CEN*) is reported in this study.
(XLSX)

S6 File Database of Ty element query sequences. Fasta file of LTR and internal sequences for Ty1-Ty5 from *S*. *cerevisiae* and Ty3p and Tsu4 from *S*. *paradoxus* used for RepeatMasker-based annotation of Ty elements in yeast genomes.
(TXT)

S7 File BED files of Ty element coordinates. Strain-specific BED12 files of Ty elements for all strains in the expanded dataset. Each Ty element in the BED12 file was categorized structurally with a prefix as full-length (f, internal region present and total length >95% of canonical length), truncated (t, internal region present and total length <95% of canonical length), or solo LTRs (s, LTR present but no match to internal region). Sequence IDs correspond to International Nucleotide Sequence Database Collaboration records except for *S*. *jurei* NCYC 3962 which uses IDs from an assembly provided by personal communication from the Daniela Delneri lab (University of Manchester).
(ZIP)

S8 File Full-length Ty1 element nucleotide sequences. Multiple sequence alignment of full-length Ty1 elements in the expanded dataset plus the canonical Ty1-H3 element (M18706). Terminal deletions exist in some sequences since Ty1 elements are classified as being full-length on the basis of having partial or complete sequences for both LTR sequences plus an internal region sequence that is >95% the length of internal region for the query Ty1 element.
(TXT)
