# Supplementary figures and images for: Evolution of Ty1 copy number control in yeast by horizontal transfer and recombination
Source: PLoS Genet. 2020 Feb 21;16(2):e1008632. doi: 10.1371/journal.pgen.1008632 (PMC7055915; doi:10.1371/journal.pgen.1008632)

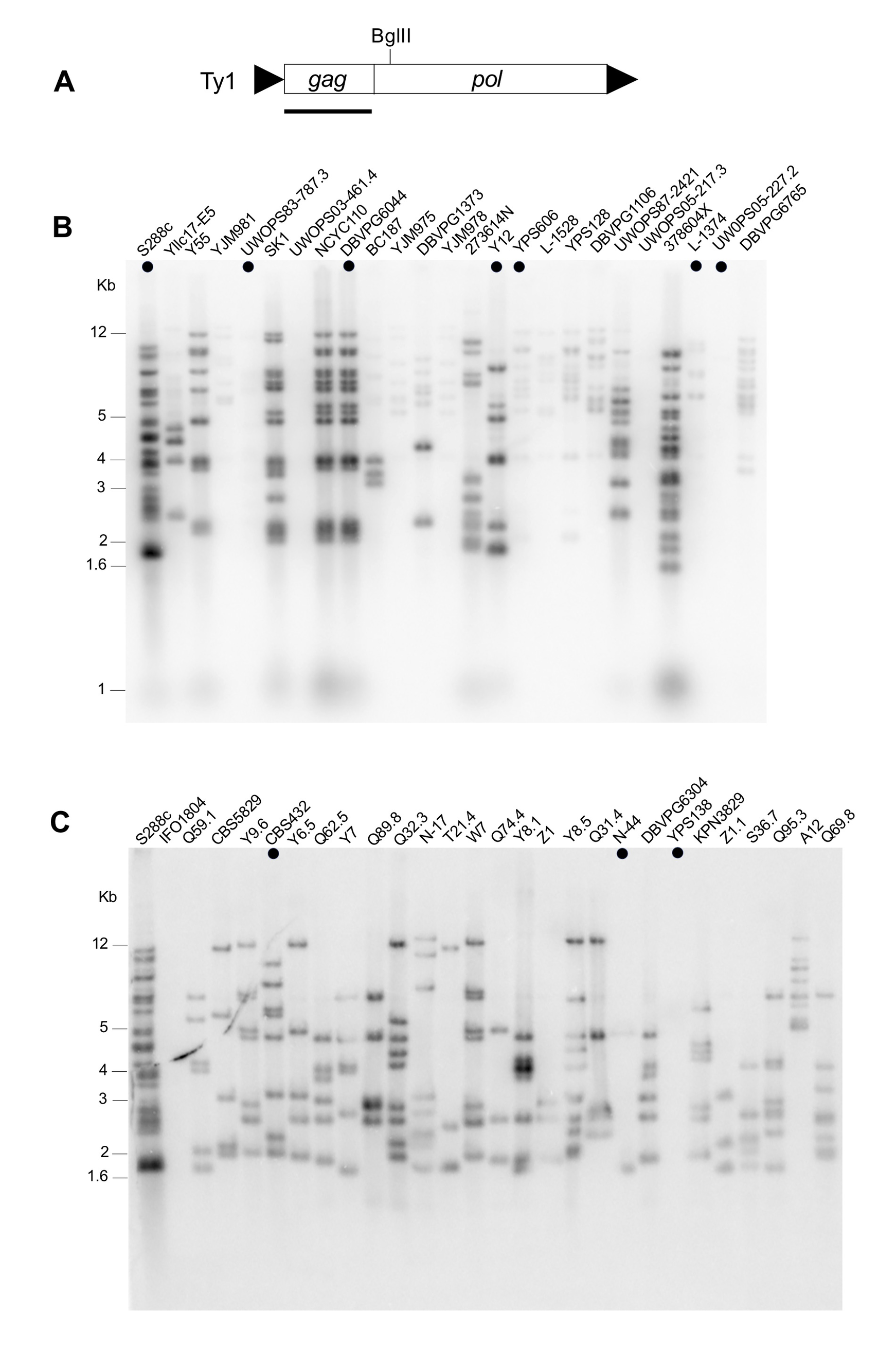

Supplement: S1 Fig — (A) Schematic of the Ty1 element, showing the gag and pol open reading frames (rectangles) and LTRs (arrowheads). The radiolabeled probe used for Southern blots is obtained from the gag gene (underlined). The location of the BglII restriction site within Ty1-H3 pol is shown above the schematic. The complete Ty1-H3 element is 5918 bp in length and gag probe is 1,162 bp in length. Southern blot results for (B) S. cerevisiae and (C) S. paradoxus. Dots in panels (B) and (C) represent strains that were selected for Ty1 mobility assays. (TIF) [file pgen.1008632.s001.tif]

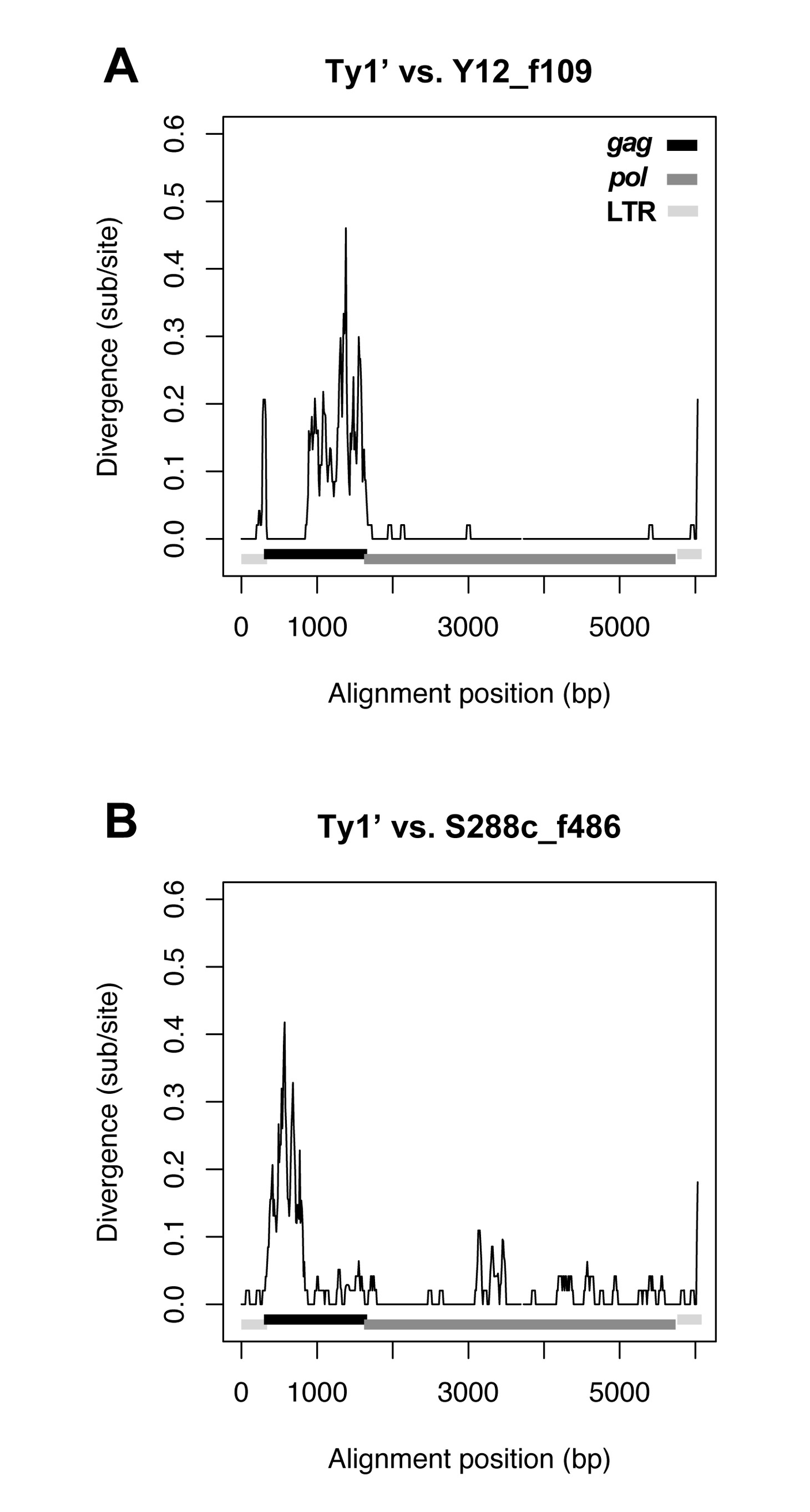

Supplement: S2 Fig — Sliding window analysis of pairwise sequence divergence between (A) Y12_f109 vs. Ty1’ and (B) S288c_f486 vs. Ty1’. The pure Ty1’ element Y12_f208 is used in both panels. Coordinates shown are relative to the multiple sequence alignment and are therefore the same for all panels. Divergence measured in substitutions per site was calculated using a Kimura 2-parameter model in overlapping 50 bp windows with a 10 bp step size. (TIF) [file pgen.1008632.s002.tif]

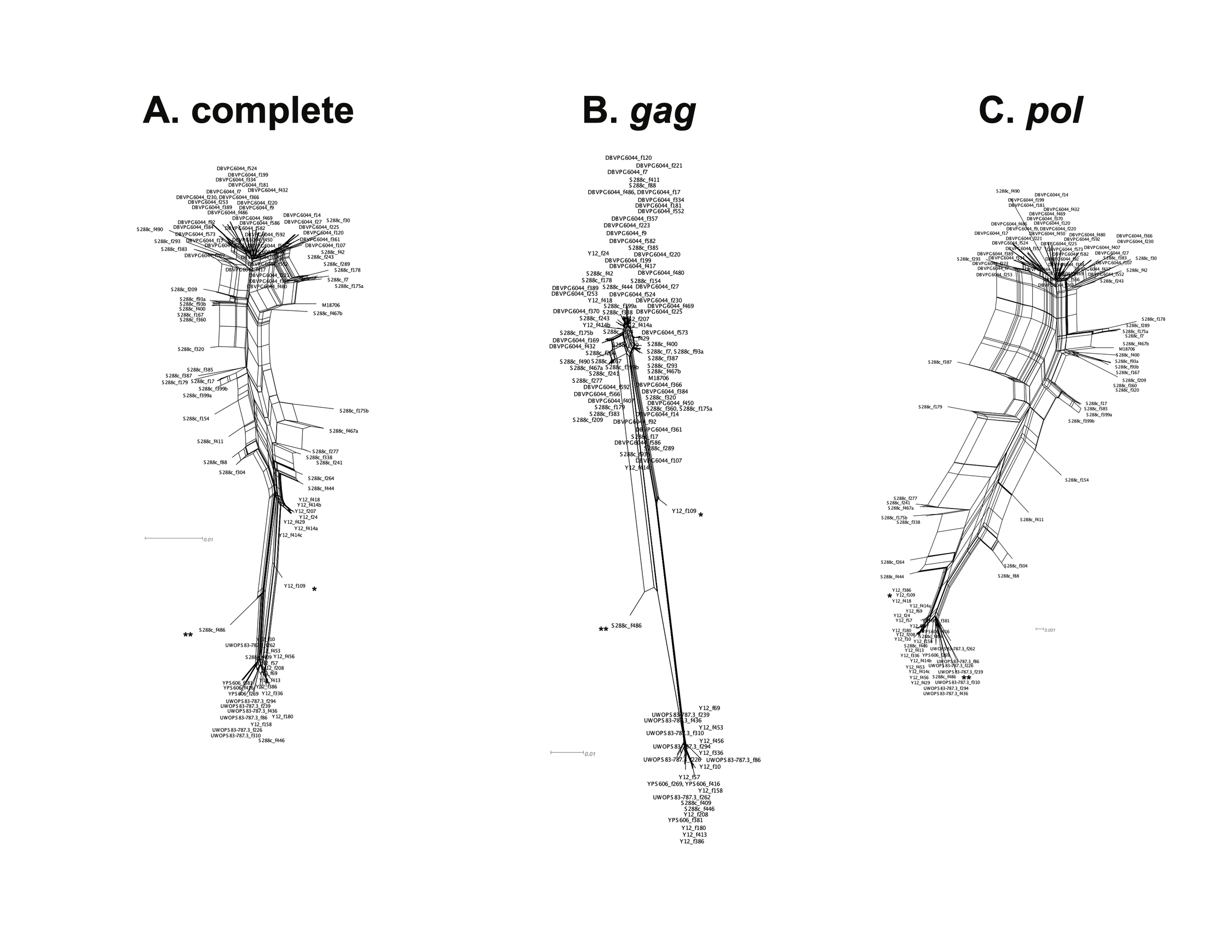

Supplement: S3 Fig — Neighbor-Net phylogenetic networks using uncorrected P-distances were constructed using (A) complete sequences, (B) gag sequences, or (C) pol sequences from full-length Ty1 elements. Nodes where incompatible partitions of sequence variation (“splits”) occur in the data because of recombination between canonical Ty1 and Ty1’ are connected by multiple edges. Bands of parallel edges would collapse to individual branches if no conflicting splits due to recombination existed in the data. Recombinant elements between Ty1’ and canonical Ty1 within gag are starred (*Y12_f109; **: S288c_f486). (TIF) [file pgen.1008632.s003.tif]

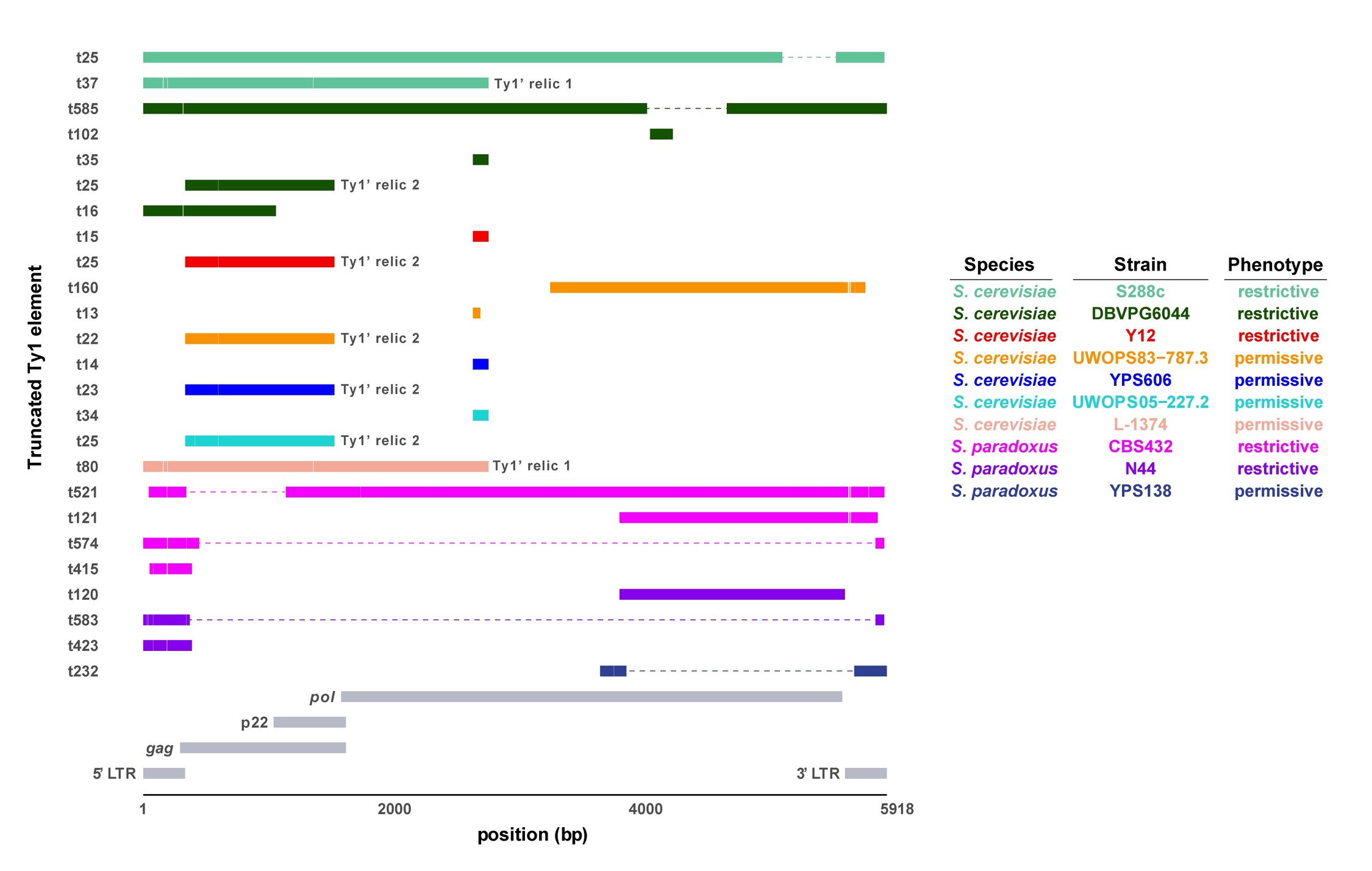

Supplement: S4 Fig — Schematic representation of regions of canonical Ty1-H3 element retained in individual truncated elements in strains with Ty1-H3 mobility data. Truncated elements are defined as having some non-LTR internal region of Ty1 present but have a total length that is <95% of the canonical Ty1 element. Strains with full-length elements are labelled in the same colors as in Fig 2. Fragments of the same truncated element are connected by dashed lines. Truncated elements labelled as Ty1’ relics were previously reported by Bleykastens-Grosshans et al. [14]. (TIF) [file pgen.1008632.s004.tif]

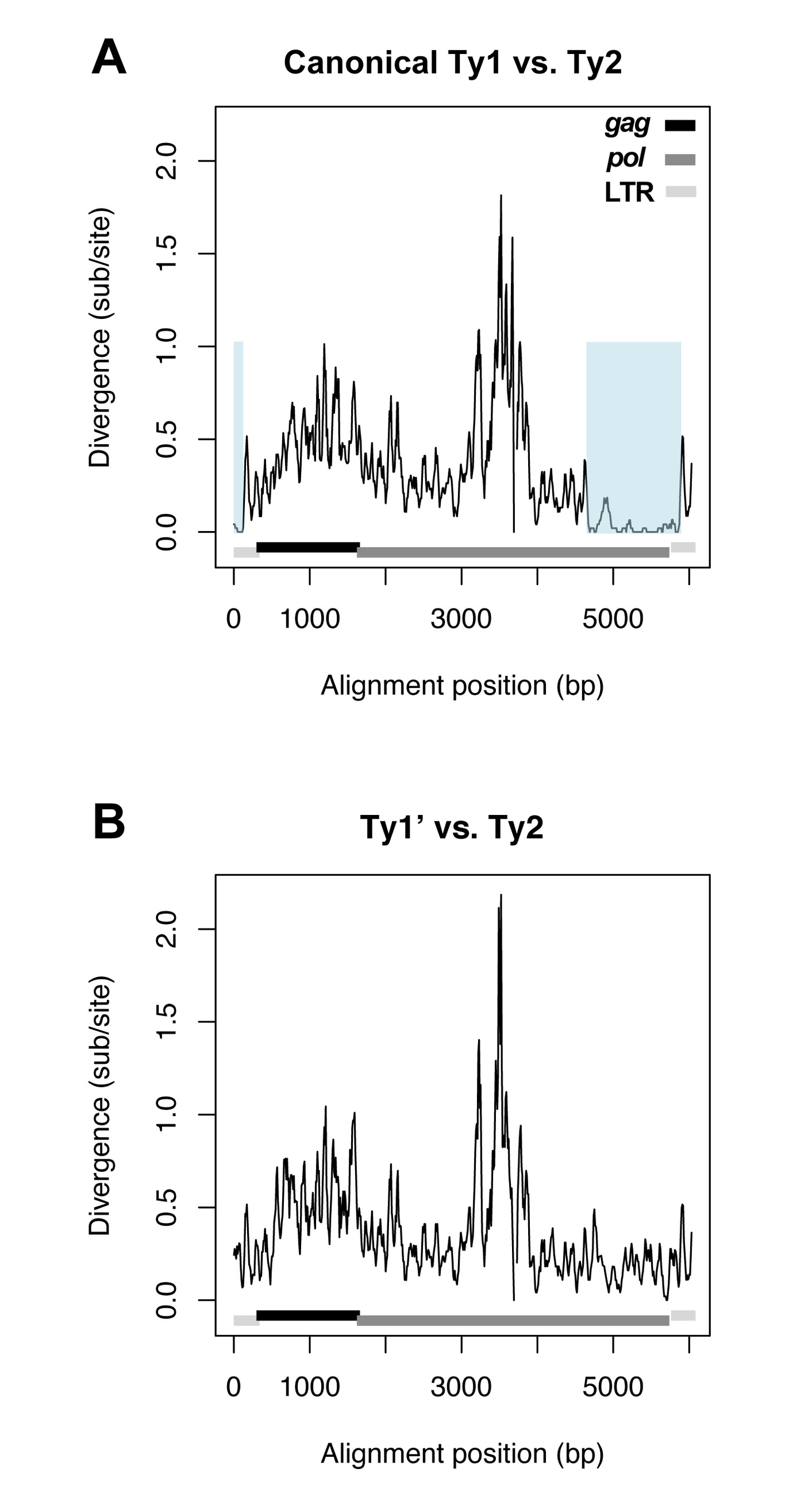

Supplement: S5 Fig — Sliding window analysis of pairwise sequence divergence between (A) canonical Ty1 vs. Ty2, and (B) Ty1’ vs. Ty2. Similarity between a subset of Ty1 elements and Ty2 in parts of the LTRs pol was previously reported in [8,23] and proposed to have arisen by recombination between these Ty families. Recombination between Ty1 and Ty2 must have occurred on an ancestor of the canonical Ty1 subfamily since high divergence between canonical Ty1 and Ty1’ in the LTRs and 3’ region of pol (blue, Fig 3A) spans the same regions that have high similarity between canonical Ty1 and Ty2 (blue, S5A Fig) but have high divergence between Ty1’ and Ty2 (S5B Fig). Identifiers for elements shown are: RepBase TY2#LTR/Copia (Ty2), DBVPG6044_f486 (pure canonical Ty1); Y12_f208 (pure Ty1’). Divergence measured in substitutions per site was calculated using a Kimura 2-parameter model in overlapping 50 bp windows with a 10 bp step size. (TIF) [file pgen.1008632.s005.tif]

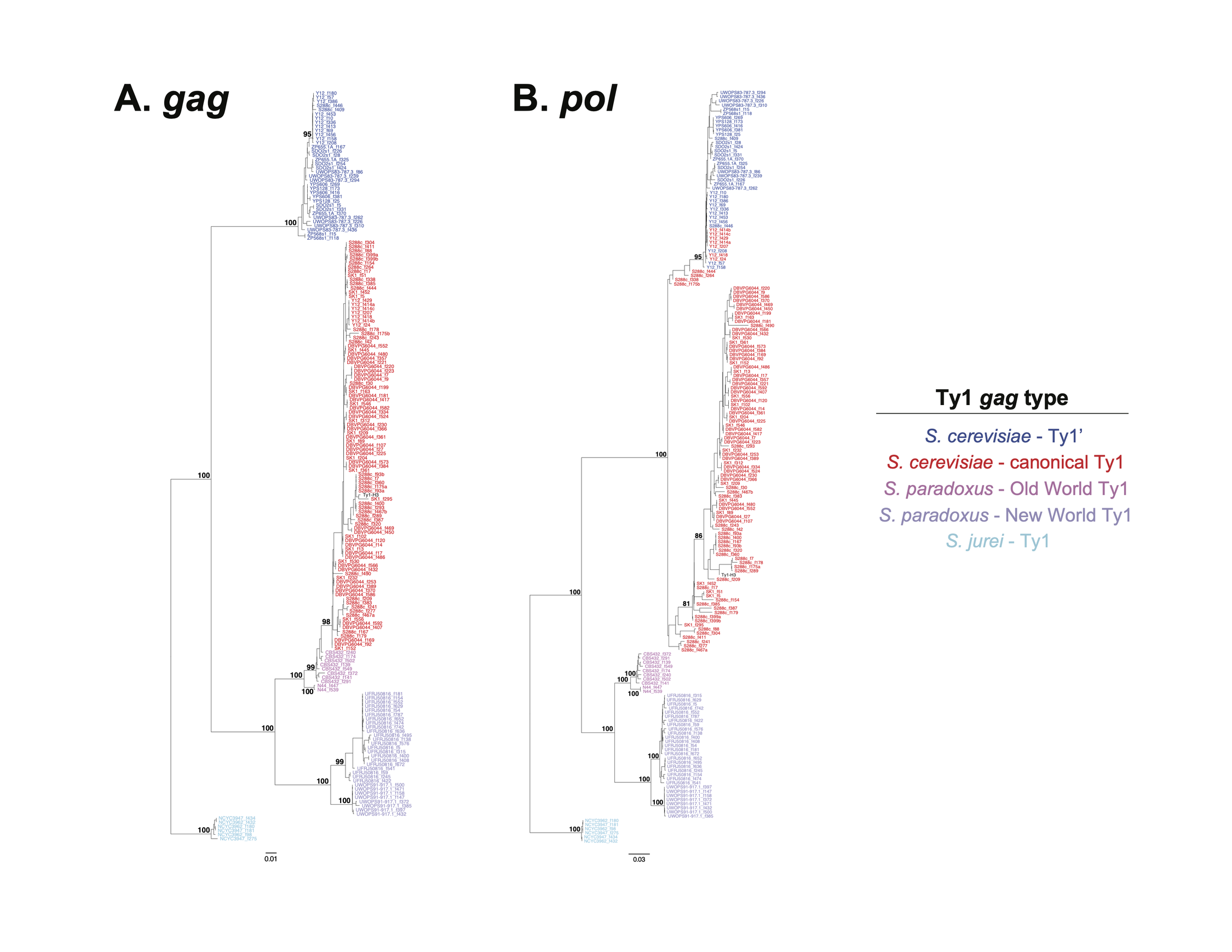

Supplement: S6 Fig — Maximum likelihood phylogenies of (A) gag and (B) pol genes from full-length Ty1 elements in complete PacBio assemblies from 15 strains of S. cerevisiae and S. paradoxus, plus two strains of the outgroup species S. jurei. The scale bar for branch lengths is in units of substitutions per site. Nodes labelled by asterisks in Fig 4 are shown with bootstrap support values. Ty1 element identifiers are a composite of strain name followed by a strain-specific unique numerical identifier prefixed by “f” indicating that it is a full-length element. Tree files in Newick format for gag and pol can be found in S2 File and S3 File, respectively. Aligned fasta sequences for all full-length Ty1 elements can be found in S8 File. (TIF) [file pgen.1008632.s006.tif]

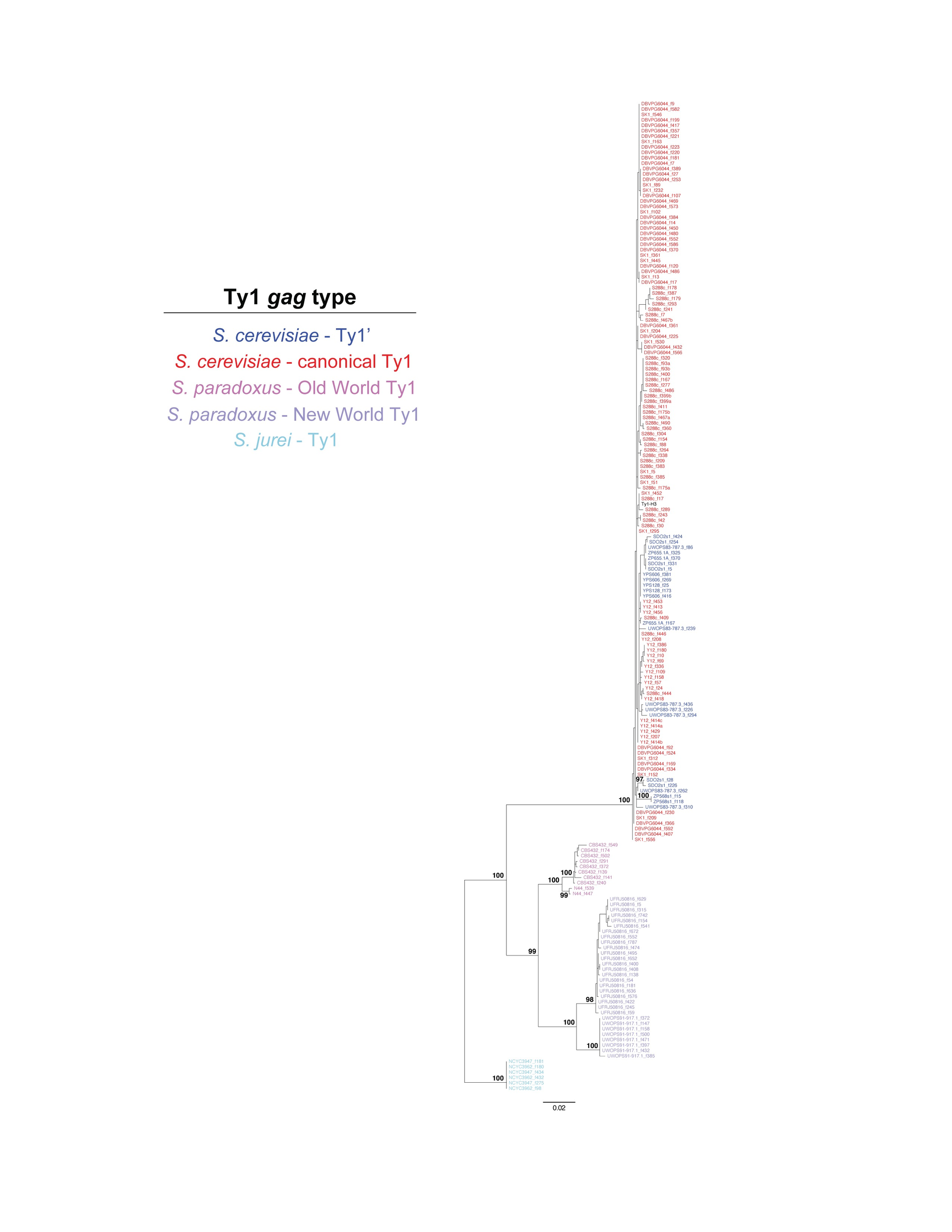

Supplement: S7 Fig — Maximum likelihood phylogenies of region of pol gene outside regions of recombination (nucleotides 1700–3000 in M18706) between canonical Ty1 and either S. paradoxus Ty1 or Ty2 from full-length Ty1 elements in complete PacBio assemblies from 15 strains of S. cerevisiae and S. paradoxus, plus two strains of the outgroup species S. jurei. The scale bar for branch lengths is in units of substitutions per site. Bootstrap support is shown for nodes with values >95%. Ty1 element identifiers are a composite of strain name followed by a strain-specific unique numerical identifier prefixed by “f” indicating that it is a full-length element. Tree file in Newick format for the non-recombinant region of pol can be found in S4 File. Aligned fasta sequences for all full-length Ty1 elements can be found in S8 File. (TIF) [file pgen.1008632.s007.tif]
